# Bioceramic bone tissue-engineered substitutes with anti-inflammatory effects in periodontitis

**DOI:** 10.3389/fbioe.2026.1934456

**Published:** 2026-09-04

**Authors:** Boying Xiao, Yuzhu Han, Chuqiao Wei, Yangfan Pei, Yunxiao Wu, Zhibo Wang, Hanchi Wang, Yanmin Zhou

**Affiliations:** 1 Department of Oral Implantology, Hospital of Stomatology, Jilin University, Changchun, China; 2 Jilin Provincial Key Laboratory of Tooth Development and Bone Remodeling, Hospital of Stomatology, Jilin University, Changchun, China

**Keywords:** antibacterial, bioceramic bone tissue, inflammation, periodontitis, signaling pathway

## Abstract

Periodontitis is a chronic inflammatory oral disease characterized by irreversible alveolar bone resorption, which severely impairs oral health and even leads to tooth loss. Clinical bone augmentation therapy for alveolar bone defects mainly relies on exogenous bone substitute materials. However, traditional materials are prone to implantation failure due to the persistent inflammatory microenvironment and bacterial infection in the periodontal area. The addition of antibiotics to improve antibacterial properties not only induces bacterial resistance but also causes systemic toxic and side effects, making it difficult to meet clinical treatment needs. This review focuses on the core demand for bone substitute materials in periodontitis treatment to simultaneously achieve antibacterial, anti-inflammatory, and osteogenic functions—an essential characteristic that distinguishes such materials from conventional antibacterial drugs and single-function bone graft materials. We systematically elaborate on multiple interrelated inflammatory signaling pathways (e.g., RANKL/RANK/OPG, cGAS-STING, NF-κB, JAK-STAT, MAPK, PI3K/AKT, HIF-1, TGF-β/SMAD, Wnt/β-catenin, Hippo) and inflammasome mechanisms involved in periodontitis-associated alveolar bone resorption, exploring potential targets for screening excellent anti-inflammatory and osteogenic active molecules. On this basis, we summarize modification strategies for bioceramic bone tissue-engineered substitutes, including incorporating metal ions (Ag, Cu, Sr, Mn, Mg, Zn, etc.) and natural anti-inflammatory molecules into the material matrix to enhance their multifunctional properties while maintaining favorable physical and biological characteristics. We also discuss structural and functional optimization of composites via surface morphology modification, photothermal, and photodynamic coating construction to improve adaptability to the periodontal inflammatory microenvironment. These reconstructed multifunctional composite bioceramic materials integrate antibacterial, anti-inflammatory, and osteogenic functions, eliminating periodontal bacterial infection, alleviating local chronic inflammation, and actively promoting osteoblast differentiation and alveolar bone defect repair, thereby perfectly matching the pathological characteristics of periodontitis. This review clarifies the core design concept of multifunctional integration for bioceramic bone tissue-engineered substitutes in periodontitis treatment and provides a theoretical basis and technical reference for developing novel bone graft materials with clinical transformation potential.

## 1 Introduction

Periodontitis is a chronic inflammatory disease that damages alveolar bone tissue and is characterized by gingival inflammation, periodontal pocket formation, alveolar bone resorption, and tooth loosening and displacement. It has emerged as the most prevalent cause of tooth loss among adults (Ha and Choung, 2020). It significantly impairs the quality of life and health of the human body. In clinical treatment, filling with exogenous bone substitute materials is usually used to achieve the effect of bone augmentation at the defect site of alveolar bone. However, due to the inflammatory environment and bacterial infection problems of patients with periodontitis themselves, the materials commonly employed in clinical practice, such as hydroxyapatite, β-tricalcium phosphate, bioceramic materials, and hybrid biphasic calcium phosphate materials, often encounter with treatment failures due to subsequent infections and inflammation after implantation. According to research. It is an effective means to improve the success rate of incremental bone surgery by modifying and treating traditional bone replacement materials to give them more antibacterial and anti-inflammatory properties. Nowadays, the utilization of implant materials to ameliorate the condition of the alveolar bone has become the main therapeutic approach. There have been an increasing number of researches and attempts regarding how to enhance bone replacement materials to replace the conventional addition of antibiotics. As the killing effect of antibiotics on bacteria within the human body also promotes the resistance of bacteria to antibiotics, an increasing number of antibiotics have lost their effective antibacterial capacity against bacteria (Okeke et al., 2024). Owing to the toxic and side effects of antibiotics, the applicable population and dosage of antibiotics are restricted (Hawking, 1952; Vannotti, 1958; Rosenberg et al., 2020; Gross et al., 2021). Severe bacterial infections can lead to severe subsequent inflammation and even failure of bone grafting (Nisyrios et al., 2020). Therefore, the development of effective antibiotic alternatives to achieve antibacterial and thus reduce inflammation is an inevitable trend in the face of the global challenge of bacterial resistance. With the development of traditional Chinese medicine, natural anti-inflammatory drugs have come to the public’s attention. Compared with non-steroidal anti-inflammatory drugs and glucocorticoids, natural anti-inflammatory drugs have a greater burden on the liver, gastrointestinal tract and kidney, resulting in side effects such as gastrointestinal ulcers, liver and kidney damage, *etc.* A large number of studies have shown that natural anti-inflammatory drugs show a gentler effect and greatly reduce the metabolic burden on various parts of the body. Moreover, studies have shown that some natural anti-inflammatory drugs, such as resveratrol, can reduce blood cholesterol and triglyceride levels, inhibit platelet aggregation, and thus reduce the risk of cardiovascular disease, which shows great advantages compared with the side effects of non-steroidal anti-inflammatory drugs and glucocorticoids on blood pressure and lipids. Metal ions are also widely used as antibacterial agents to inhibit inflammation because of their diverse antibacterial mechanisms, high stability *in vivo*, broad antibacterial spectrum, high safety and resistance. At the same time, compared with the addition of foreign substances with anti-inflammatory effects in the material, it is also a new treatment idea to treat the commonly used bone replacement materials themselves to achieve stable and efficient antibacterial and anti-inflammatory effects, including the surface shape treatment design combined with bionics, such as spiky, brush, bowl, *etc.* Plasma treatment to prepare bioactive coating, combined with photothermal therapy and photodynamic therapy to modify the material, and when achieving efficient antibacterial effect, the bone replacement material combined with photothermal therapy and photodynamic therapy has a stronger promotion effect on the differentiation and proliferation of osteoblasts, which is conducive to the development of bone, antibacterial, and osteoblast. Anti-inflammatory multifunctional materials have great guiding significance. The synthesis of the above preparation and reconstruction ideas has certain guiding significance for the treatment of bone defects.

## 2 Molecular regulatory mechanisms in periodontitis

Inflammation is a complex and reversible process involving multiple signaling pathways that are interconnected and interfere with each other. Natural anti-inflammatory drugs can block one or more of these links to achieve resistance to inflammation. For the future modification and selection of materials, we can follow the way from micro-regulation to phenotypic change and explore potential excellent anti-inflammatory drugs from the internal action mechanism of drugs. The following section systematically elaborates core inflammatory signaling cascades and osteogenic regulatory mechanisms, establishing a clear mechanistic framework for the subsequent screening and functional verification of bioactive anti-inflammatory molecules.

### 2.1 Inflammatory signaling pathways

#### 2.1.1 cGAS-STING signaling pathway

In mammalian cells, the detection of foreign DNA is an important part of immunity, a process performed by the stimulator of cyclic GMP-AMP synthase (cGAS) of the interferon gene (STING) pathway (Ablasser and Chen, 2019). As a molecular messenger pathway, it involves two proteins: cyclic guanosine adenylate synthase synthase (cGAS) and the stimulator of interferon gene (STING). This process involves the binding of cGAS to double-stranded DNA (dsDNA) to catalyze its own activity, producing a second messenger molecule and a potent agonist for STING, 2′3′ cyclic GMP-AMP (cGAMP) (Decout et al., 2021). This signaling molecule then activates the STING protein located in the endoplasmic reticulum; Subsequently, the STING signaling pathway triggers an immune response that induces the expression of a range of genes associated with inflammation (Decout et al., 2021). For example, cGAS-STING stimulation also promotes inflammation by triggering p52-RELB nuclear translocation to promote a nonclassical NF-κB response (Bakhoum et al., 2018; Hou et al., 2018).

In periodontitis, invasive periodontal pathogens (e.g., Porphyromonas gingivalis, *Fusobacterium* nucleatum) and damaged host cells release cytoplasmic bacterial DNA and host genomic dsDNA, which robustly activate the cGAS-STING pathway in gingival epithelial cells, macrophages and periodontal ligament cells. Persistent cGAS-STING activation promotes abundant secretion of pro-inflammatory cytokines, accelerating gingival inflammatory infiltration and aggravating periodontal tissue destruction. Targeted inhibition of this cascade is expected to limit excessive inflammatory response, which offers a potential direction for developing anti-inflammatory biomaterials for periodontal regeneration (Liu et al., 2025).

Mechanism-guided molecular screening: The cGAS-STING pathway serves as a critical innate immune sensor in periodontal inflammation. Several natural bioactive molecules summarized in Section 3.2 can specifically modulate STING-mediated immune activation and downstream inflammatory cascades, providing feasible natural targeted intervention strategies for cGAS-STING-driven periodontal inflammatory injury (Navashin et al., 1977).

**FIGURE 1:**
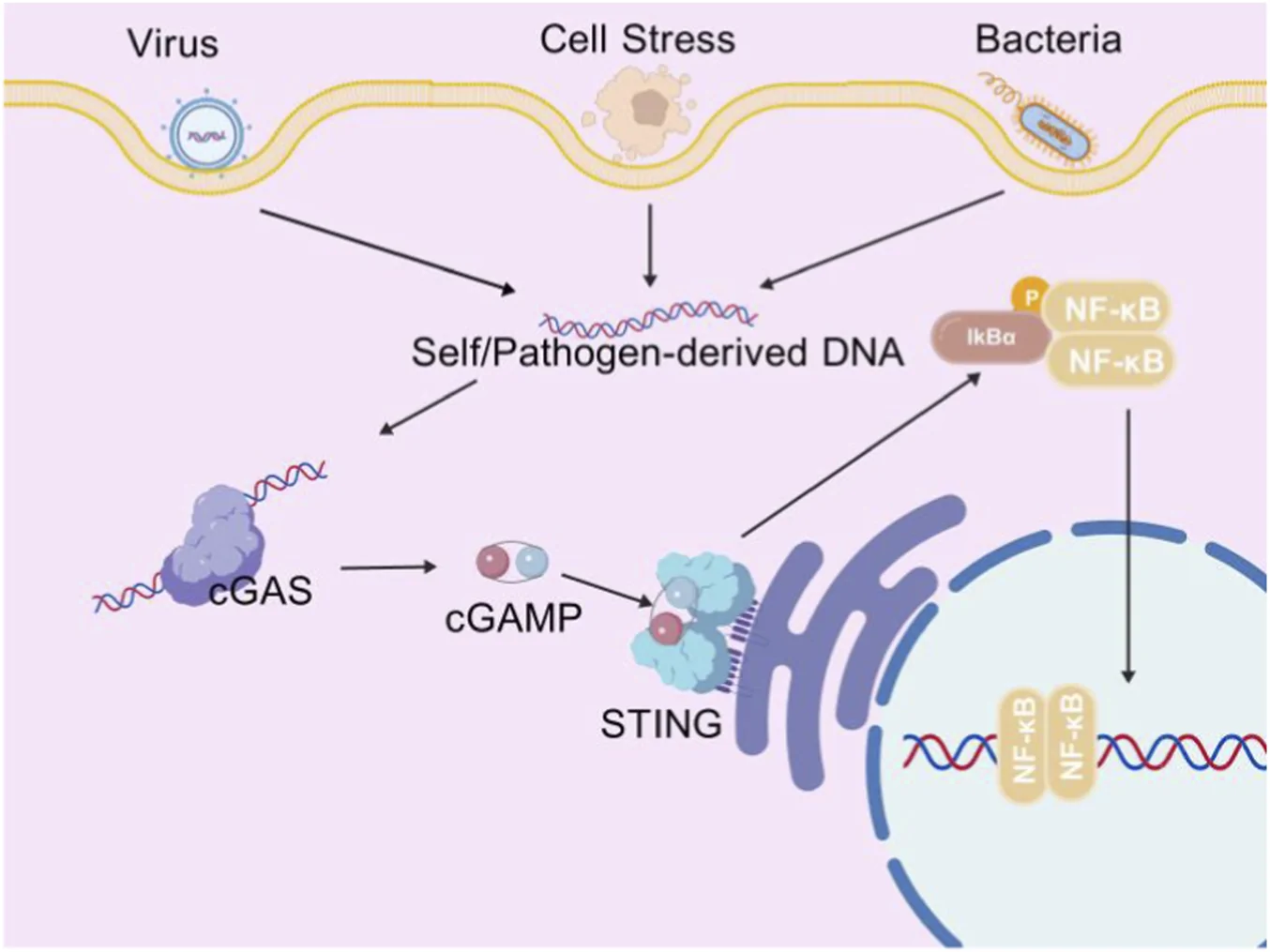
cGAS-STING signaling pathway, binding of cGAS to double-stranded DNA (dsDNA) to catalyze its own activity, to produce cGAMP. Then cGAMP activates the STING protein. Created with BioGDP.com.

For a schematic overview of this pathway, see Figure 1.

#### 2.1.2 NF-κB signaling pathway

NF-κB is a family of inducible transcription factors that regulate a large number of genes involved in immune and inflammatory response (Oeckinghaus and Ghosh, 2009). It consists of Rel (c-Rel), RelA (p65), RelB, p50/p105 (NF-κB1), and p52/p100 (NF-κB2) (Zhang et al., 2016). The activation of NF-κB involves two major signaling pathways, the classical and nonclassical (or alternative) pathways. The nonclassical NF-κB pathway selectively responds to a specific group of stimuli, including ligands from a subgroup of TNFR superfamily members, such as LTβR, BAFFR, CD 40, and RANK. The classical NF-κB pathway responds to different stimuli, including ligands for various cytokine receptors, pattern recognition receptors (PRR), members of the TNF receptor (TNFR) superfamily, as well as T-cell receptors (TCR) and B-cell receptors. Functionally, classical NF-κB is involved in almost all aspects of the immune response, whereas the nonclassical NF-κB pathway appears to have evolved as a complementary signaling axis that cooperates with the classical NF-κB pathway in the regulation of specific functions of the adaptive immune system (Liu et al., 2017). Stimulatory factors activate signaling molecules, including the IKK complex, that phosphorylate and degrade the phosphorylation and degradation of IκB proteins, thereby releasing and activating the NF-κB complex (Taniguchi and Karin, 2018). The primary mechanism of typical NF-κB activation is the triggering of the inducible degradation of IκBα through site-specific phosphorylation of the multisubunit IκB kinase (IKK) complex. IKK consists of two catalytic subunits, IKKα and IKKβ, and a regulatory subunit known as the NF-κB essential regulator (NEMO) or IKKγ. Upon activation, IKK phosphorylates IκBα at both N-terminal serines, thereby, triggering ubiquitin-dependent IκBα degradation in the proteasome, leading to rapid and transient nuclear translocation of typical NF-κB members (mainly p50/RelA and p50/c-Rel dimers). The nonclassical NF-κB pathway does not involve IκBα degradation but relies on the processing of the NF-κB2 precursor protein p100. The pathway IKKα mediates the phosphorylation of p100, which in turn induces p100 ubiquitination and processing, p100 processing involves the degradation of its C-terminal Iκ B-like structure, resulting in the production of mature NF-κB2 p52 and the nuclear translocation of the nonclassical NF-κB complex p52/RelB nuclear translocation (Karin and Ben-Neriah, 2000; Hayden and Ghosh, 2004; Liu et al., 2017).

In periodontal lesions, pathogen-derived virulence factors potently activate classical NF-κB in resident periodontal cells and infiltrated immune cells, driving massive production of TNF-α, IL-1β and IL-6. Meanwhile, noncanonical NF-κB signaling is closely linked to RANKL transcription, which facilitates osteoclastogenesis and pathological alveolar bone resorption. The dual roles of NF-κB in inflammation and bone loss make it a central therapeutic target for periodontitis (Wu et al., 2025).

Mechanism-guided molecular screening: As the core hub of periodontal inflammatory response and osteoclastic bone resorption, the NF-κB pathway is the most common target of natural anti-inflammatory molecules. Multiple natural compounds described in Section 3.2, including paeoniflorin, ginkgolide B (Zhang et al., 2011), triptolide, berberine and resveratrol (Bhattarai et al., 2016), exert anti-inflammatory and bone-protective effects *via* targeted inhibition of NF-κB cascade activation, verifying the feasibility of pathway-directed molecular selection.

**FIGURE 2:**
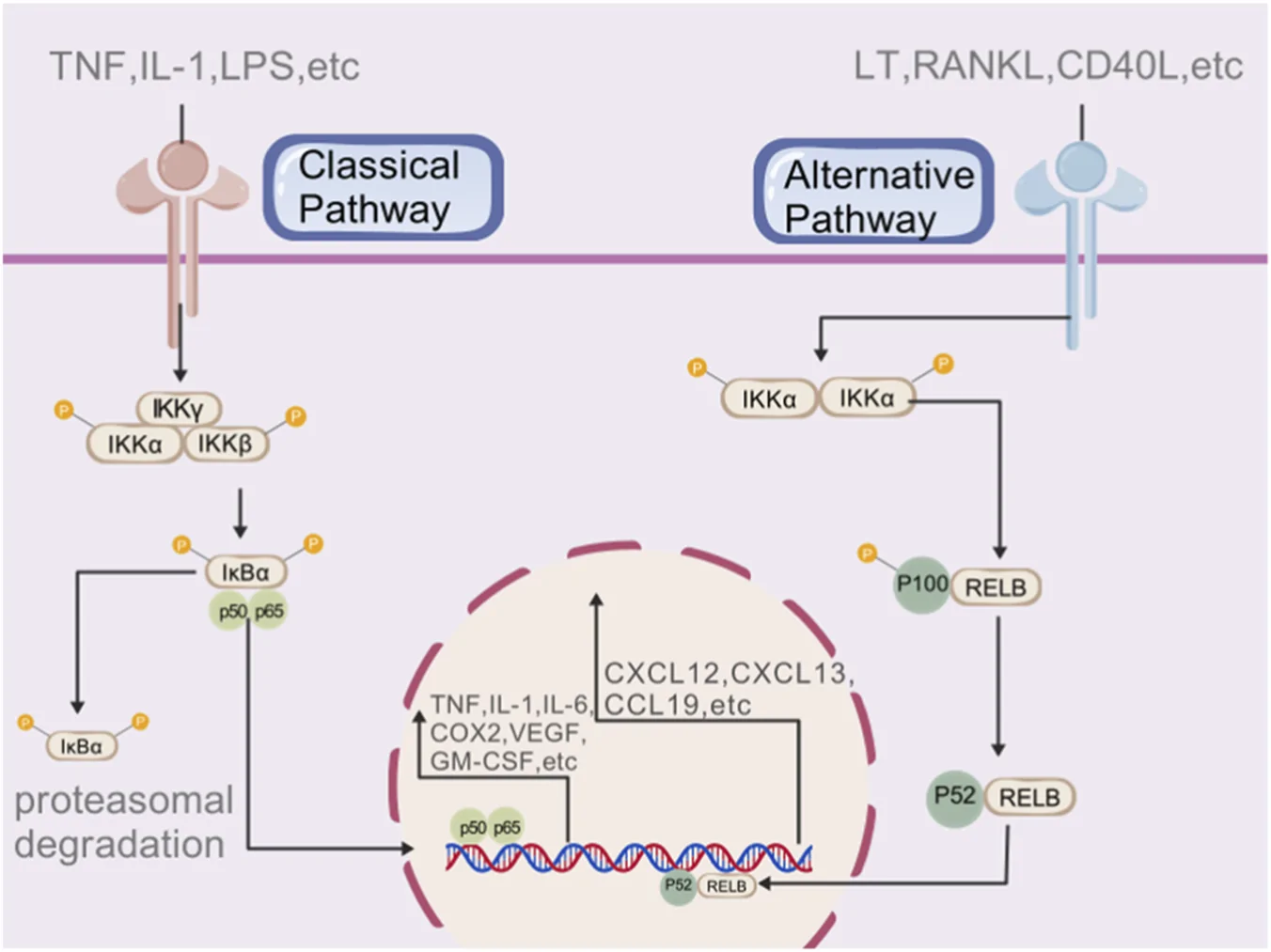
Schematic diagram of the NF-κB signaling pathway. Distinct stimuli activate different receptors, triggering separate signaling cascades that ultimately regulate target gene transcription. Created with BioGDP.com.

A schematic overview of the classical and alternative NF-κB signaling pathways is shown in Figure 2.

#### 2.1.3 JAK-STAT signaling pathway

The JAK/STAT pathway consists of three major components: tyrosine kinase-associated receptors that receive signals. Tyrosine kinases that transmit signals. Transcription factors of the JAK generating effector pathway. There are four types of JAK, namely, JAK 1, JAK 2, JAK 3 and TYK 2. Different JAK-dependent cytokine receptors signal through different JAKs. Each receptor consists of multiple subunits, each of which binds to a JAK (Clark et al., 2014). For example, the common γ-chain (γc) used by IL-2, IL-4, IL-7, IL-9, IL-15, and IL-21 binds only to JAK 3 and is the only receptor subunit using JAK 3 (Hofmann et al., 2002). The JAK 3 family of cytokine receptors is composed of seven subunits. There are seven members of the mammalian STAT family: STAT 1, STAT 2, STAT 3, STAT 4, STAT 5A, STAT 5B, and STAT 6. Upon activation of the JAK-associated cytokine receptors, cytoplasmic STAT undergoes tyrosine phosphorylation and dimerization and translocates to the nucleus. Each member of the STAT family can be activated by a variety of cytokines and their associated JAK activation (O’Shea et al., 2015).

In periodontitis, abundant pro-inflammatory cytokines within periodontal pockets, including IL-6, IL-17 and IFN-γ, persistently activate JAK-STAT signaling. STAT3 and STAT1 are the most extensively investigated isoforms in periodontal tissues: activated STAT3 amplifies inflammatory circuits and upregulates RANKL expression in periodontal ligament cells, whereas STAT1 mediates Th1-type immune responses. Regulating JAK-STAT activity can suppress excessive immune infiltration and relieve inflammatory destruction of periodontal tissues (Saliem et al., 2022).

Mechanism-guided molecular screening: Persistent JAK-STAT3 activation aggravates periodontal inflammation and bone loss. Natural molecules such as astaxanthin, paeoniflorin, and andrographolide (Lee et al., 2011) can effectively inhibit JAK-STAT signaling hyperactivation, serving as ideal natural regulators targeting immune-inflammatory cascades in periodontitis.

**FIGURE 3:**
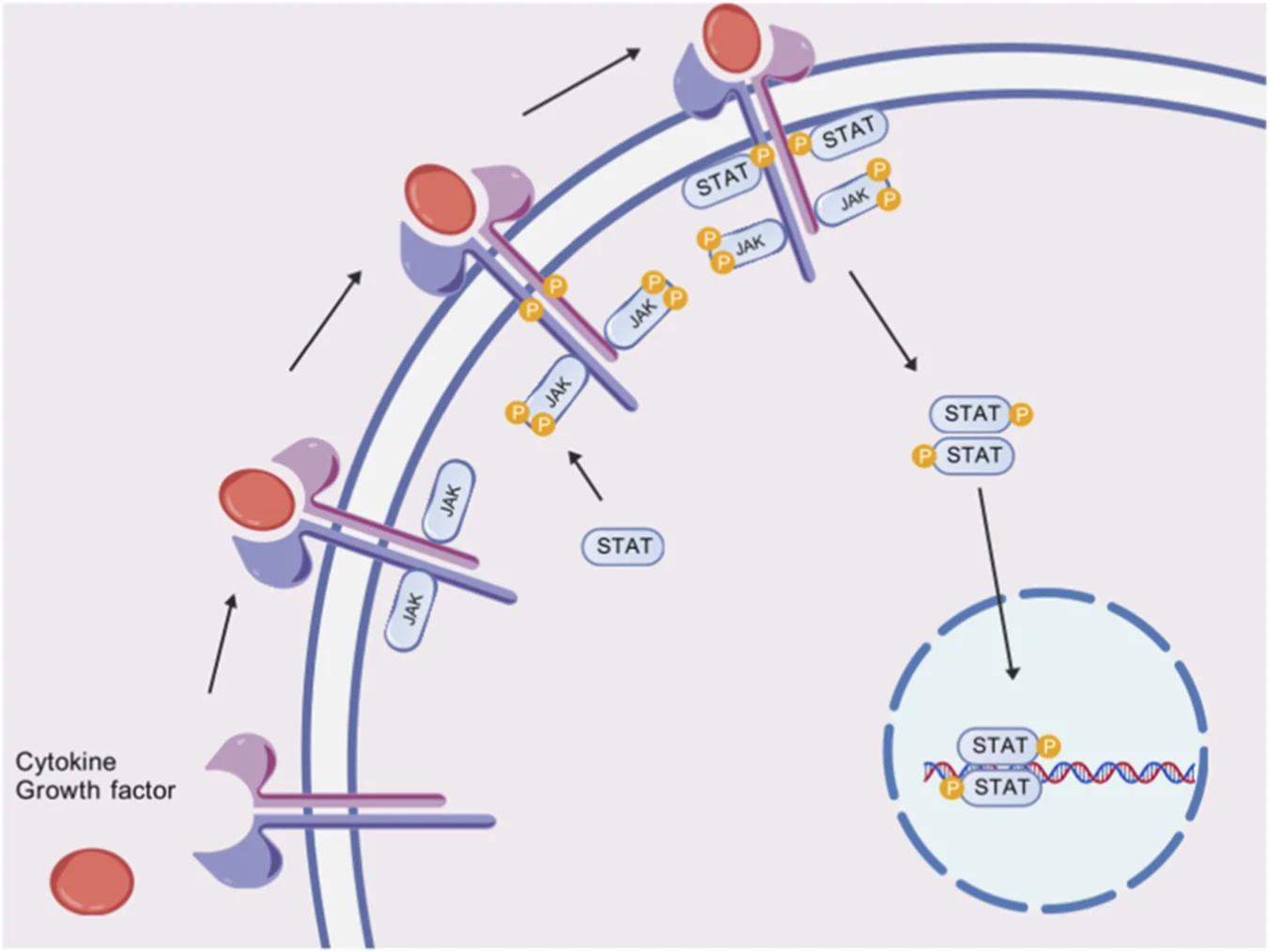
JAK-STAT signaling pathway. The cytokine actives the JAK, then cytoplasmic STAT undergoes tyrosine phosphorylation and dimerization and translocates to the nucleus. Created with BioGDP.com.

A schematic illustration of the JAK-STAT signaling pathway is presented in Figure 3.

#### 2.1.4 MAPK signaling pathway

The MAP kinase family consists of four major subfamilies of related proteins (extracellularly regulated kinase 1/2 (ERK 1/2), c-Jun N-terminal kinase (JNK), p38, and extracellularly regulated kinase 5 (ERK 5)).

The activation of this signaling pathway mainly consists of the following steps: extracellular signaling → membrane receptor → RAS → MAP3K → MAP2K → MAPK → downstream target genes (Cicenas et al., 2018). The first MAP kinase network discovered was the (RAS-RAF-ERK) signaling cascade, defined by ERK 1 and ERK 2 (Campbell et al., 1995; Seger and Krebs, 1995). The ERK cascade plays a role in cell proliferation, differentiation, and survival, and in many cases ERK activity is dependent on mutations in RAS and RAF kinases (Cicenas et al., 2017a, 2017b). The c-Jun N-terminal kinase cascade has been shown to be a major component of the cell proliferation and differentiation process. The c-Jun N-terminal kinases (JNK) are a subfamily of mitogen-activated protein (MAP) kinases, in which there are three differently spliced genes, JNK 1, JNK 2, and JNK 3. Abnormal activation of JNK is found in many cancers, as well as in inflammatory and neurodegenerative diseases. p38 is another subfamily consisting of four isoforms: α, β, γ, and δ(Zarubin and Han, 2005). Pathogens or inflammatory stimuli initiate a cascade of reactions mediated by p38 kinases. Mitogen-activated protein kinase kinase five-extracellular regulated kinase 5 (MEK 5-ERK 5) cascade. Activation of this pathway is a common event in tumor development, and it is involved in anti-apoptotic signaling and chemoresistance (Drew et al., 2012).

In periodontitis, LPS and bacterial virulence factors robustly activate p38 and JNK cascades in gingival fibroblasts and macrophages. Activated MAPK signaling synergizes with NF-κB to boost pro-inflammatory cytokine secretion, impair periodontal ligament cell viability, and indirectly promote osteoclastic alveolar bone resorption. Inhibiting MAPK overactivation is a promising strategy to mitigate inflammatory damage in periodontal lesions (de Molon et al., 2026).

Mechanism-guided molecular screening: The MAPK pathway acts as a synergistic amplifier of periodontal inflammation. Multiple natural anti-inflammatory molecules including astaxanthin, paeoniflorin (Zhou et al., 2024), ginkgolide B and ginsenoside exert anti-inflammatory and bone-protective functions by blocking ERK/JNK/p38 MAPK cascades, providing multi-target intervention options for inflammatory signal amplification.

**FIGURE 4:**
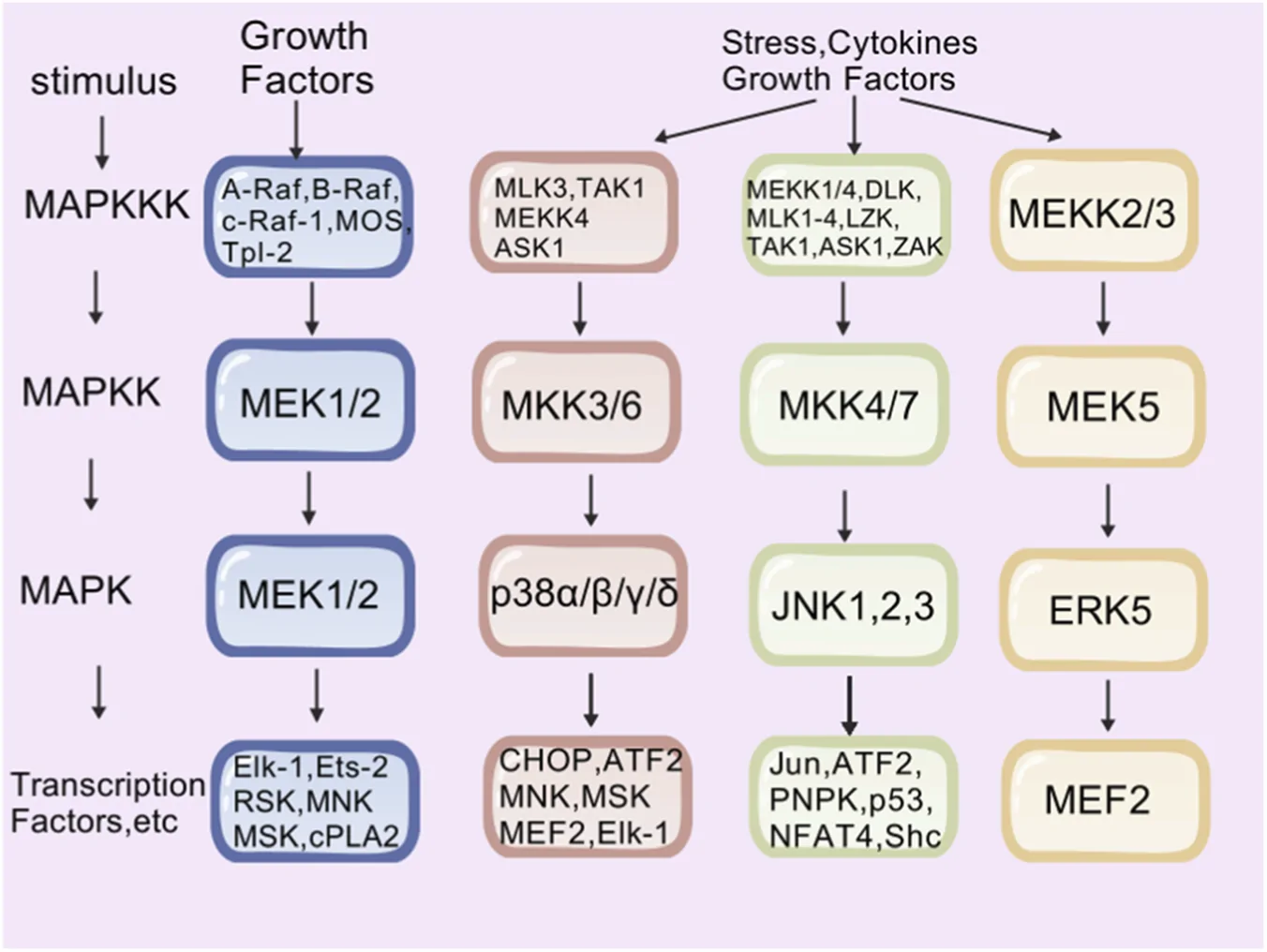
MAPK signaling pathway. The stimulus activate the signaling pathway by the following steps: extracellular signaling → membrane receptor → RAS → MAP3K → MAP2K → MAPK → downstream target genes. Created with BioGDP.com.

A schematic diagram of the MAPK signaling pathway is shown in Figure 4.

#### 2.1.5 PI3K/AKT signaling pathway

PI3K is a lipid kinase and a downstream effector of G protein-coupled receptor (GPCR) and receptor tyrosine kinase (RTK). Phosphatidylinositol (PI) is a specific type of membrane lipid that can be phosphorylated to yield phosphatidylinositol-4-phosphate (PIP) and phosphatidylinositol-4,5-bisphosphate (PIP2). Activated PI3K phosphorylates PIP2 to generate phosphatidylinositol-3,4,5-trisphosphate (PIP3) which acts as a second messenger involved in the signaling process. There are three isoforms of PI3K, type I, type II, and type III (Wang et al., 2022).

Akt is a serine/threonine-specific protein kinase with three isoforms, Akt1, Akt2, and Akt3, encoded by the protein kinase α, protein kinase β, and protein kinase γ (PKBα, PKBβ, and PKBγ) genes, respectively. It consists of an N-terminal homology structural domain, a kinase structural domain, and a C-terminal regulatory structural domain. Activated Akt regulates cell growth, proliferation, cell cycle, and glucose metabolism by further phosphorylating glycogen synthase kinase-3β (GSK-3β), mTOR, endothelial nitric oxide synthase (eNOS), FoxO3a, nuclear factor-κB (NF-κB), p21 (Ediriweera et al., 2019), glucose metabolism, and other physiological functions (Ediriweera et al., 2019; Zhang and Zhang, 2019). mTOR is an important downstream target of the PI3K/Akt signaling pathway (Weichhart, 2017) and an important regulator of autophagy, and exists in two different complexes, of which mTOR complex 1 (mTORC1) is a core component downstream of PI3K/Akt (Kwiatkowski, 2003). The mTOR complex is an essential component of the PI3K/Akt signaling pathway.

Activated Akt (p-Akt) directly activates mTORC1 by phosphorylating the Ser2448 site on mTORC1, while p-Akt phosphorylates mTORC2, and phosphorylated mTORC2 in turn fully activates Akt, which in turn activates the PI3K/Akt/mTOR signaling pathway to regulate downstream autophagy (Wanting et al., 2023). Tensin homolog (PTEN), tuberous sclerosis complex (TSC) 1 (hamartin) and TSC2 (tuberin) can inhibit mTOR activity. The mechanism by which PTEN inhibits PI3K/AKT/mTOR signaling pathway is that PTEN can reverse PIP 3 to PIP 2 to avoid AKT activation. Akt phosphorylation of TSC 2 releases its inhibitory effect on mTOR. When AKT is phosphorylated, It inhibits the phosphorylation of TSC 2 in the dimer protein complex TSC 1/TSC 2, leading to activation of phosphorylation of mTORC 1 (p-mTOR) (Porta et al., 2014; Jin et al., 2023).

The PI3K/Akt signaling pathway plays a negative regulatory role in lipopolysaccharides (LPS)-induced inflammation, and inhibition of the PI3K/Akt pathway upregulates the expression of NF-κB, TNF-α, and IL-6 in monocytes, exacerbating the inflammatory response (Arab et al., 2021; Yan et al., 2022; You et al., 2022). In periodontal tissues, intact PI3K/Akt signaling protects periodontal ligament stem cells against inflammatory oxidative stress and supports osteogenic capacity. Therefore, sustaining proper PI3K/Akt activity is beneficial for alleviating inflammation and preserving regenerative potential of periodontal progenitor cells (Saliem et al., 2022; Wu et al., 2025).

Mechanism-guided molecular screening: Distinct from pro-inflammatory cascades, the PI3K/AKT pathway exerts protective and regenerative effects in periodontitis. Natural molecules such as astaxanthin (Zhang et al., 2021c), paeoniflorin and berberine can activate PI3K/AKT signaling to resist inflammatory damage and enhance osteogenesis, representing a protective targeted therapeutic strategy different from inflammatory pathway inhibition.

**FIGURE 5:**
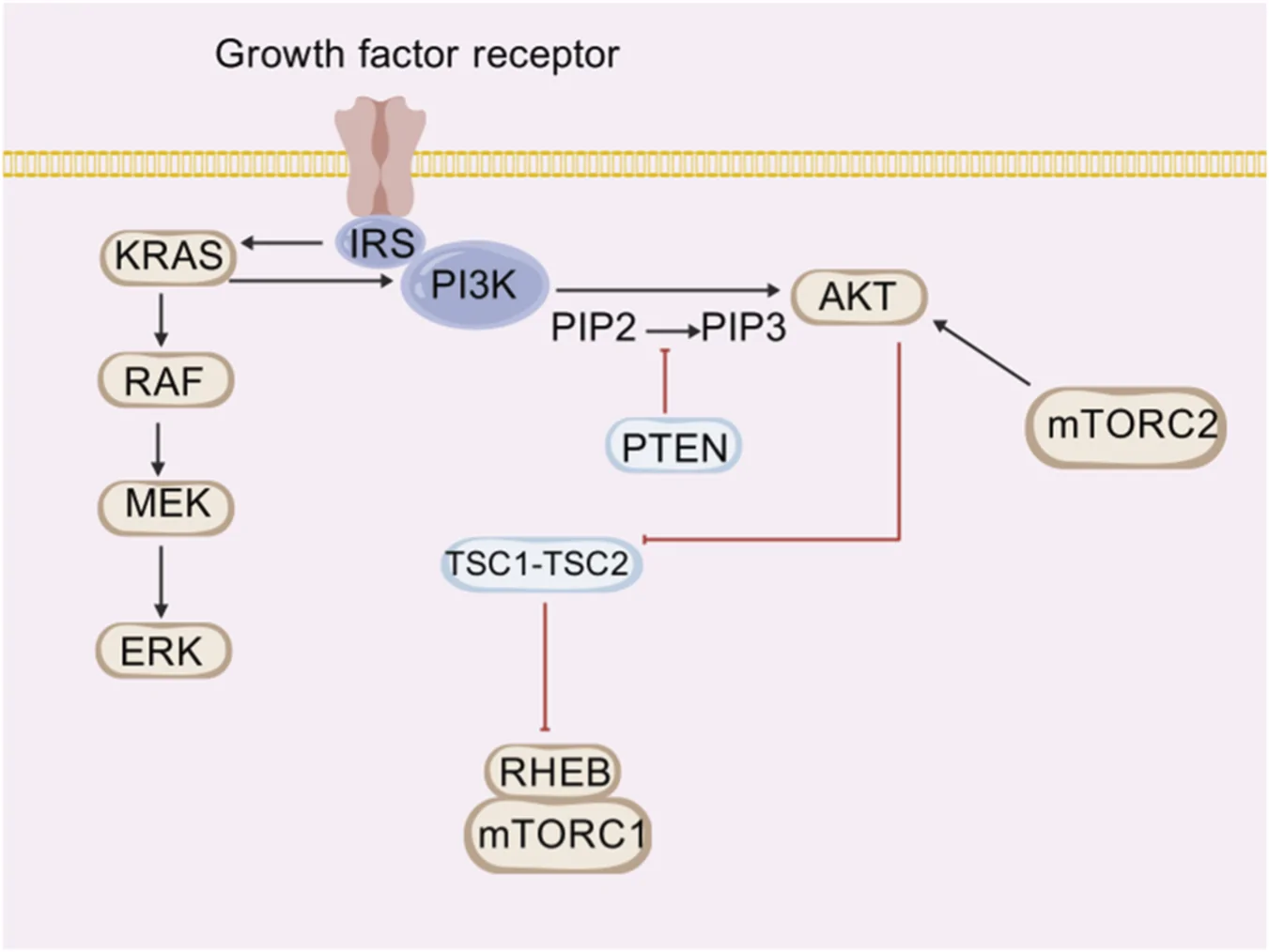
PI3K/AKT signaling pathway. After stimulation of receptor tyrosine kinases (RTKs) and G protein-coupled receptors, phosphatidylinositol 4,5-bisphosphate (PIP2) is converted into phosphatidylinositol 3,4,5-trisphosphate (PIP3). PIP3 can bind to the N-terminal PH domain of protein kinase B (PKB, Akt). This activates Akt. Activated Akt can directly phosphorylate mTOR or maintain the GTP-bound state of Rheb (an important small GTPase) through TSC2, and then enhance the activation of mTOR. Created with BioGDP.com.

A schematic overview of the PI3K/AKT signaling pathway is presented in Figure 5.

#### 2.1.6 Vesicle

Inflammatory vesicles are currently classified into five main groups: the NOD-like receptor thermal protein domain associated protein 1 (NLRP 1) inflammatory vesicles, the NLRP 3 inflammatory vesicles, the NLRC 4 inflammatory vesicles, the pyrin inflammatory vesicles (Spel et al., 2022), and the absent in melanoma 2 (AIM2) inflammatory vesicles. The three main components of the NLRP 3 inflammatory vesicles are NLRP 3, which captures danger signals and recruits downstream molecules; cysteine asparaginase-1, which facilitates the maturation of the cytokines IL-1β and IL-18 as well as the gasdermin D processing to mediate cytokine release and pyroptosis; and ASC (apoptosis-associated speck-like protein containing the cysteine recruitment domain, which functions as a bridge connecting NLRP 3 and cysteine-1 (Fu and Wu, 2023). NLRP 3 nucleates the assembly of inflammatory vesicles, leading to cytokine-mediated protein hydrolysis activation by cysteine-1β (IL-1β) family cytokines. Mediated activation of protein hydrolysis and induction of inflammatory, pyroptotic cell death (Mangan et al., 2018).

Mechanism-guided molecular screening: NLRP3 inflammasome-mediated pyroptosis is a key driver of aggravated periodontal inflammation. Multiple natural antioxidants including EGCG, quercetin (Di Cristo et al., 2022) and resveratrol can effectively suppress NLRP3 inflammasome activation and subsequent inflammatory pyroptosis, targeting the terminal inflammatory amplification link of periodontitis.

#### 2.1.7 Autophagy mechanism

Autophagy mechanisms are initiated under stressful conditions, such as hypoxia, starvation, exposure to toxic molecules, or other reticular stresses, to maintain cellular homeostasis and adapt to unfavorable environments. Impaired autophagy has been shown to cause inflammation among the gut (Gordon and Martinez, 2010). mTOR is a highly conserved serine/threonine protein kinase consisting of two forms of complexes called mTOR complex 1 (mTORC1) and mTOR complex 2 (mTORC2). mTORC1 activation is implicated in a variety of complex signaling pathways, such as Nod-2-mediated tolerance and the production of anti-inflammatory cytokines, including the downstream target ribosomal protein S6 (RPS6). S6 (RPS6). It has been shown that silencing mTOR significantly attenuates lipopolysaccharide (LPS)-induced inflammation and oxidative damage, as opposed to exacerbating inflammation and oxidative damage by the genetically inactivated autophagy control factor ATG 5 (Perl, 2018). As seen in the study, LPS activates mTOR and inhibits autophagy in experimental colitis through the upstream toll-like receptor 4 (TLR4) signaling pathway, while mTOR-dependent autophagy coordinates its downstream NF-κB activation, leading to the production of proinflammatory cytokines and oxidative stress (Zhou et al., 2018).

mTOR plays a central role in sensing metabolic stress and promoting growth and proliferation by inhibiting autophagy in most types of mammalian cells, regulating the activation of the TLR 4-MyD 88 MAPK signaling pathway and the NF-κB pathway (Perl, 2018).

Mechanism-guided molecular screening: Autophagy dysfunction mediated by mTOR hyperactivation exacerbates periodontal inflammatory injury. Natural molecules including sinomenine (Li et al., 2023) and resveratrol can restore impaired autophagy and inhibit mTOR overactivation, thereby alleviating periodontal inflammation *via* autophagy regulation.

#### 2.1.8 HIF-1 signaling pathway

The hypoxia-inducible factor 1 family is a major regulator of the cellular response to hypoxia, including two subunits, α and β, which play important roles in angiogenesis, cellular energy metabolism, wound healing, inflammatory response, and fibrotic diseases (Cummins et al., 2016). HIF-1α is a major regulator of the cellular response to hypoxia, including the α and β subunits.

HIF-1α also enhances inflammatory response by activating nuclear factor κB, which promotes phagocytosis by macrophages and neutrophils and enhances the activity of immune cells (Atri et al., 2018). HIF-1α and nuclear factor κB have several common target genes, co-regulators, and co-stimulators, so in different microenvironments, they can either promote or inhibit each other (Zhang et al., 2019) and have similar functions, e.g., HIF-1α and nuclear factor κB have similar functions. HIF-1α and nuclear factor-κB both regulate macrophage polarization (Xie et al., 2022).

Biological signaling pathways are intricately interconnected, and dysregulation of the LPS signaling pathway, the TGFβ/SMAD signaling pathway (Saika et al., 2016), the Wnt/β-catenin signaling pathway (Vallée and Lecarpentier, 2018), and the hippo (Wang H. et al., 2023) signaling pathway all mediate inflammation, and are interconnected with each other.

### 2.2 Osteogenic signaling pathway

The alveolar bone undergoes a continuous process of bone remodeling and osteogenesis. An imbalance in alveolar bone homeostasis due to inflammation will ultimately result in the resorption of the alveolar bone. There are multiple cellular synergies in the progression of periodontitis, with osteoclasts and osteoblasts being the direct executors of alveolar bone resorption and formation, respectively. Other cellular participants in alveolar bone homeostasis include osteoblasts, periodontal ligament cells, macrophages, monocytes, neutrophils, and adaptive immune cells (Li Y. et al., 2021). The synergistic effects of multiple signaling pathways gathered among different cells interact to determine whether there is homeostasis or imbalance in osteogenesis and bone remodeling. For example,: immune cells promote bone destruction by inducing expression of nuclear factor-κB ligand receptor activator (RANKL) in osteoblasts and periodontal ligament cells; Neutrophils contribute to bone destruction in periodontitis by activating the OSMR/RL-D4/RANKL axis in osteoblasts (Ando et al., 2024); Periodontal membrane stem cells, a type of odontogenic MSCs, have osteogenic differentiation potential and can be applied to regenerative repair of periodontal tissues (Ha and Choung, 2020); Bone marrow mesenchymal stem cells (BMSCs) are subjected to mechanical sensitivity, especially tensile strain, and can differentiate into osteoblasts. This contributing bone property is facilitated by Wnt, BMP, Notch, MAPK, PI3K/Akt, and Hedgehog (Hh) signaling pathway (Zhang et al., 2024a); Osteoclasts are also involved in osteogenesis. Since osteoclasts also secrete exosomes that drive osteogenic differentiation of marrow mesenchymal stem cells (MSCs), they promote osteogenic differentiation of MSCs by cleaving phosphoprotein 1 (SPP1) through activation of TGFβ1/SMAD3 signaling (Faqeer et al., 2023). RANKL binds to RANK to induce osteoclast differentiation and promote bone resorption. OPG competitively binds to RANK with RANKL and promotes bone formation. Receptor activator for nuclear factor-κB ligand/receptor activator for nuclear factor-κB/osteoprotegerin (RANKL/RANK/OPG) system is the final link in alveolar bone remodeling (Zaidi et al., 2023).

#### 2.2.1 The RANKL/RANK/OPG axis: final common pathway of alveolar bone remodeling

The receptor activator of nuclear factor-κB ligand/receptor activator of nuclear factor-κB/osteoprotegerin (RANKL/RANK/OPG) system (Zhang et al., 2025) constitutes the final and indispensable regulatory link in alveolar bone remodeling, serving as the central molecular checkpoint that integrates upstream pro-inflammatory and pro-resorptive signals to control osteoclastogenesis and bone resorptionecrosis factor (TNF) superfamily, is expressed as both a membrane-bound and a soluble form by osteoblast lineage cells, periodontal ligament cells, activated T lymphocytes, and osteocytes. Upon binding to its cognate receptor RANK, which is expressed on the surface of osteoclast precursors and mature osteoclasts, RANKL triggers a cascade of downstream intracellular signaling events—including activation of NF-κB, MAPK (ERK, JNK, p38), and NFATc1 pathways—that drive the differentiation, fusion, activation, and survival of osteoclasts. In the periodontal inflammatory milieu, elevated levels of pro-inflammatory cytokines such as TNF-α, IL-1β, and IL-6 further upregulate RANKL expression in stromal and immune cells, amplifying osteoclastogenic signaling and tipping the balance toward bone destruction (Valverde et al., 2025).

OPG, a soluble decoy receptor also belonging to the TNF receptor superfamily, is secreted primarily by osteoblasts and stromal cells and functions as the physiological counterweight to RANKL. By competitively binding to RANKL, OPG prevents RANKL from engaging RANK on osteoclast precursors, thereby inhibiting osteoclast differentiation, reducing mature osteoclast activity, and promoting osteoclast apoptosis. The relative ratio of RANKL to OPG in the local bone microenvironment is thus the principal determinant of osteoclastogenic activity and net bone turnover. In periodontitis, inflammatory mediators not only increase RANKL production but also suppress OPG expression, creating a markedly elevated RANKL/OPG ratio that drives excessive osteoclast formation and progressive alveolar bone loss. Genetic and pharmacological studies have confirmed the non-redundant role of this tripartite system: OPG-deficient mice develop severe osteoporosis due to unrestrained osteoclast activity, whereas RANKL ablation or OPG overexpression leads to osteopetrosis characterized by impaired osteoclastogenesis. Therapeutically, agents that block RANKL signaling (such as the monoclonal antibody denosumab) have demonstrated efficacy in reducing bone resorption across multiple osteolytic conditions, underscoring the centrality of this axis in bone homeostasis regulation (Ozaki et al., 2017).

#### 2.2.2 Signaling pathways governing osteogenic differentiation

In parallel to the osteoclastogenic RANKL/RANK/OPG axis, a network of evolutionarily conserved signaling pathways governs the osteogenic differentiation of mesenchymal progenitor cells and the functional activity of mature osteoblasts (Dang et al., 2024), collectively determining the bone-forming capacity of the periodontium (Zhao et al., 2023).

The Wnt/β-catenin (canonical Wnt) pathway is a master regulator of MSC fate commitment toward the osteoblastic lineage. Wnt ligands binding to Frizzled and LRP5/6 co-receptors inhibit β-catenin degradation, allowing its nuclear translocation and activation of TCF/LEF transcription factors that drive the expression of osteogenic genes such as Runx2 and Osterix. Non-canonical Wnt pathways (Wnt/JNK and Wnt/Ca^2+^) also contribute to osteogenic differentiation through β-catenin-independent mechanisms. The bone morphogenetic protein (BMP) pathway, a branch of the TGF-β superfamily, signals through Smad1/5/8 transcription factors to potently induce osteoblast differentiation and bone formation; BMP-2 and BMP-7 are clinically applied to promote bone regeneration. BMP signaling intersects extensively with the Wnt pathway, and both converge on the master osteogenic transcription factor Runx2 (Liu et al., 2016).

The Notch signaling pathway plays a context-dependent role in osteogenesis: while it maintains MSC stemness and suppresses premature differentiation in some contexts, activated Notch can also promote osteoblast maturation at later differentiation stages, with its effects modulated by crosstalk with BMP and Wnt signals. The mitogen-activated protein kinase (MAPK) family—encompassing ERK, JNK, and p38 pathways—transduces extracellular stimuli including growth factors, mechanical strain, and inflammatory signals to regulate osteoblast proliferation, differentiation, and matrix mineralization. The PI3K/Akt pathway mediates survival and anabolic signals in osteoblasts and MSCs, promoting cell proliferation and enhancing osteogenic differentiation in response to growth factors such as IGF-1. Finally, the Hedgehog (Hh) pathway contributes to osteoblast differentiation during skeletal development and adult bone repair, acting upstream of both BMP and Wnt signaling to promote osteogenic lineage commitment.

#### 2.2.3 Osteoimmune crosstalk and coupling of bone resorption and formation

Bone remodeling is not simply the independent action of osteoclasts and osteoblasts but requires tightly coupled communication between these cell lineages—a process profoundly modulated by the immune system in inflammatory conditions such as periodontitis. The concept of osteoimmunology captures this intricate interface: immune cells and bone cells share regulatory molecules and signaling pathways, and chronic inflammation disrupts the physiological coupling of bone resorption and formation.

Beyond the RANKL/RANK/OPG axis, additional molecular mediators facilitate osteoblast-osteoclast crosstalk. The EphrinB2/EphB4 bidirectional signaling system (Fu et al., 2024), for example, mediates direct cell-cell contact between osteoclasts and osteoblasts: forward signaling through EphB4 in osteoblasts promotes bone formation, while reverse signaling through EphrinB2 in osteoclasts inhibits osteoclast differentiation, serving as a coupling mechanism that transitions the remodeling unit from resorption to formation (Zhou et al., 2023). Osteoclasts also release factors sequestered in the bone matrix—including TGF-β and IGFs—during resorption, which in turn recruit osteoprogenitor cells and stimulate osteoblastic bone formation at the same site.

In periodontitis, the sustained inflammatory milieu disrupts this physiological coupling. Pro-inflammatory cytokines not only drive excessive osteoclastogenesis *via* RANKL upregulation but also directly impair osteoblast function and survival, shifting the balance from coupled remodeling to uncoupled net bone loss. Macrophages, T cells, and neutrophils all contribute to this dysregulation: Th17 cells produce IL-17 which further amplifies RANKL expression; regulatory T cells and anti-inflammatory cytokines such as IL-10 and IFN-γ exert protective, anti-osteoclastogenic effects that are overwhelmed in chronic disease (de Molon et al., 2026). Understanding this multilayered signaling network is essential for identifying therapeutic targets that can restore alveolar bone homeostasis in periodontal disease.

**FIGURE 6:**
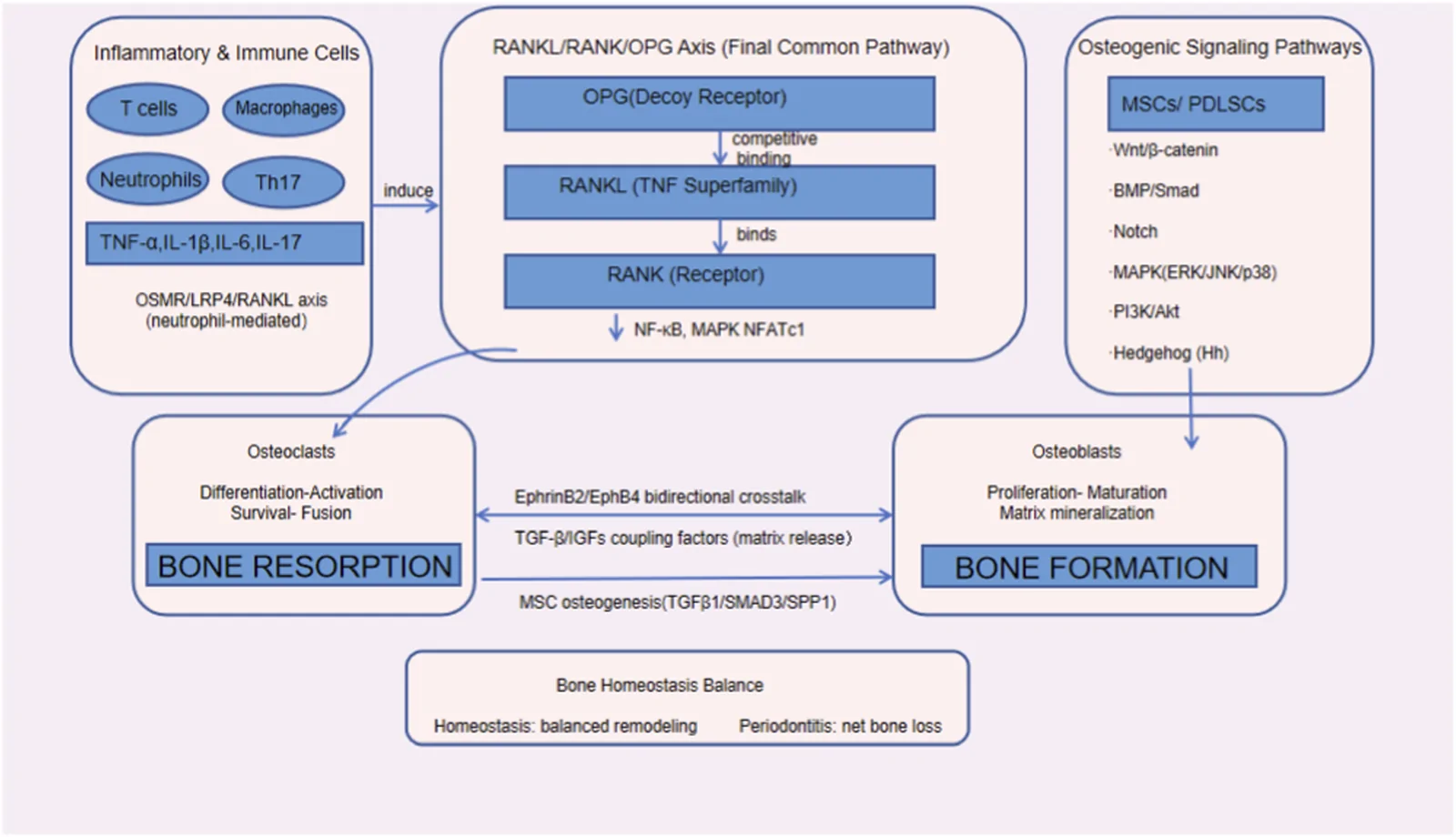
Regulatory network of bone remodeling in bone homeostasis and periodontitis. Pro-inflammatory cytokines derived from inflammatory and immune cells initiate osteoclastogenesis mainly through the RANKL/RANK/OPG signaling axis. RANKL binds to RANK and activates NF-κB/MAPK/NFATc1 pathways to promote osteoclastic bone resorption, while OPG acts as a decoy receptor to competitively inhibit RANKL. The OSMR/LRP4/RANKL axis contributes to neutrophil-mediated regulation of osteoclast activity. Meanwhile, multiple osteogenic pathways in MSCs/PDLSCs orchestrate osteoblast maturation and bone formation. Osteoclasts and osteoblasts communicate reciprocally *via* EphrinB2/EphB4, TGF-β/IGFs and TGFβ1/SMAD3/SPP1 signals to coordinate bone turnover. Under physiological conditions, bone resorption and formation remain balanced. In periodontitis, inflammatory perturbation breaks this coupled remodeling, resulting in net alveolar bone loss.

A schematic overview of the inflammatory signaling network regulating bone homeostasis is presented in Figure 6.

## 3 Bioactive anti-inflammatory molecules

Bioactive inorganic metal ions have been widely incorporated into periodontal bone repair scaffolds as multifunctional regulatory factors. They exert synergistic anti-inflammatory, antibacterial, pro-osteogenic, and pro-angiogenic effects by modulating immune response, oxidative stress, and core inflammatory signaling pathways (NF-κB, STAT3, TLRs, STING). Notably, nearly all ion-mediated biological effects are strictly concentration-dependent. The antimicrobial and anti-inflammatory mechanisms (including ROS overproduction, cell membrane disruption, protein denaturation, and metabolic inhibition) that eliminate oral pathogenic bacteria will induce cytotoxicity toward host periodontal ligament cells (PDLCs), mesenchymal stem cells (MSCs), and osteoblasts at slightly elevated concentrations. Therefore, clarifying the therapeutic window (effective and safe concentration range) of each metal ion is essential for the rational design of clinical applicable anti-inflammatory bone scaffolds.

### 3.1 Metal ions

#### 3.1.1 Strontium (Sr)

Strontium enhances the metabolic activity of osteoblasts. It inhibits osteoclast activity by decreasing anti-tartrate acid phosphatase activity and inhibiting calcium phosphate membrane resorption in a dose-dependent manner. In addition, promotes osteoblast proliferation and alkaline phosphatase activity (Gentleman et al., 2010). Strontium-doped bone substitute materials or coatings exhibit excellent long-term or short-term antimicrobial efficacy (Huang et al., 2024).

Therapeutic window and cytotoxicity risk: Excessively high Sr concentration disrupts intracellular calcium ion homeostasis and mitochondrial function in host osteoblasts and PDLCs. Although strontium has relatively low biological toxicity compared with other heavy metal ions, overdose exposure still inhibits cell proliferation and osteogenic differentiation, resulting in impaired bone regeneration. Controlled slow ion release is the key to maintaining the osteoimmunomodulatory benefits while avoiding host cell damage (Chen et al., 2025; Iwamatsu-Kobayashi et al., 2017).

#### 3.1.2 Zinc (Zn)

Zinc (Zn) has good bactericidal and anti-inflammatory properties and not only by inhibiting matrix metalloproteinase-bound MMP collagen-sensitive cleavage sites (Choi et al., 2020). It also stimulates the production of proteins by osteoblasts, controls ECM mineralization, and influences the expression of bone marker genes including osteoblastic polypeptide (OPN) and osteocalcin (OC) Increases alkaline phosphatase (ALP), a marker of osteoblast differentiation expression, and decreases the expression of bone sialic acid proteins. It has antimicrobial properties and a positive biological response (Cacciotti, 2017; Ion-doped mesoporous bioactive glass: preparation, characterization, and applications using the spray pyrolysis method, 2022). Excess extracellular Zn2+ exhibits toxic effects through inhibition of manganese uptake and disruption of carbon metabolism, and acts as a bactericidal agent based on this property (Read et al., 2019). At the same time zinc also disrupts cellular membranes and intracellular components (e.g., cytoplasmic proteins and DNA). Electrostatic and other types of interactions with bacterial membranes lead to their dysfunction. This is all related to the antimicrobial properties of zinc (Vitasovic et al., 2024).

As a cofactor for several transcription factors and enzymes zinc promotes wound healing by promoting fibroblast proliferation and epithelial cell migration (Lusvardi et al., 2009; Anand et al., 2014; Miola et al., 2015).

Zinc (including metal ions such as magnesium, silver, and copper) collectively exerts anti-inflammatory effects by reducing host oxidative stress damage, modulating immune cells, and inhibiting inflammatory signaling pathways such as nuclear transcription factor κB, Toll-like receptors, STAT3, and NOD. Zn2+ is also involved in the regulation of Cu/Zn superoxide dismutase (SOD) activity, which indirectly regulates the scavenging of intracellular ROS (Chen Y. et al., 2022).

Therapeutic window and cytotoxicity risk: Zinc exhibits a narrow effective therapeutic window. The same ROS overaccumulation and membrane destruction mechanisms that eradicate bacteria will trigger cytotoxicity in host PDLCs and MSCs at marginally elevated concentrations. Excess extracellular Zn^2+^ induces intracellular ion imbalance, mitochondrial dysfunction, and oxidative damage, significantly inhibiting cell viability and osteogenic differentiation. Therefore, excessive Zn doping will reverse the beneficial effects and impair periodontal bone regeneration, which is a key constraint for Zn-based biomaterial modification (Ganguly et al., 2022).

#### 3.1.3 Manganese (Mn)

Manganese (Mn) In addition to increasing osteoblast proliferation and viability (Miola et al., 2014), Mn ions can exhibit antimicrobial activity against a wide range of bacteria by attaching to sulfhydryl groups, causing bacterial death by binding to proteins, altering their structure, driving cell wall rupture, and ultimately inhibiting the functioning of DNA associated with bacterial division and replication, without harming the cell (Nawaz et al., 2018). Existing studies suggest that manganese ions are a newly discovered STING agonist that can be used for immune response enhancement to achieve antimicrobial utility (Jia et al., 2024).

Therapeutic window and cytotoxicity risk: High-concentration Mn exposure causes abnormal activation of the STING inflammatory pathway in host cells, triggering excessive immune inflammation and oxidative stress. Excess Mn accumulates in mitochondria of osteoblasts and PDLCs, inhibits cell metabolic activity, induces cell apoptosis, and ultimately suppresses osteogenic differentiation. The immune-enhancing effect of Mn is concentration-buffered; overdose converts immune protection into inflammatory injury, limiting its application in anti-inflammatory periodontal materials (Chen et al., 2025).

#### 3.1.4 Magnesium (Mg)

Mechanisms by which Magnesium (Mg) ions inhibit inflammation include inhibition of phagocyte activation, opening of calcium channels, activation of N-methyl-d-aspartate (NMDA) receptors, and nuclear factor (NF)-κB activation (Shahi et al., 2019). Magnesium is also involved in the regulation of Peroxisome proliferator-activated receptor γ (PPAR-γ) gene expression. PPAR-γ regulates cytokine production by inhibiting the expression of proinflammatory molecules and the activity of other transcription factors (e.g., activator protein-1 and NF-kB) (Li J. et al., 2021; Locatelli et al., 2021). At the same time, magnesium is involved in a wide variety of metabolic activities in the body and is required for many biological processes, such as protein and nucleic acid synthesis, cell cycle and cytoskeletal integrity phagocytosis is activated, and active calcium transport is regulated. and magnesium plays an important role in the bone formation phase (Dietrich et al., 2009; Ma et al., 2011; Diba et al., 2012; Tabia et al., 2019).

Therapeutic window and cytotoxicity risk: Mg has a relatively safe concentration range among all functional metal ions, but high-dose Mg release still causes adverse biological effects. Excess Mg^2+^ disturbs intracellular calcium-magnesium ion balance, inhibits osteoblast mineralization, and impairs the adhesion and proliferation of PDLCs. Excessive suppression of inflammatory signaling by high-concentration Mg also weakens the normal immune defense of periodontal tissues, reducing the ability to clear residual pathogens and hindering tissue repair (Chen et al., 2025).

#### 3.1.5 Copper (cu)

Copper (Cu) facilitates the synthesis of proangiogenic factors and osteogenic differentiation in BMSC. And the slower release rate suggests that cu could be an ideal material for long-term antimicrobial activity. The mechanism may be that the influx of excess Cu ions into bacteria induces copper-like death by killing the bacteria, inhibiting energy production, triggering oxidative stress, and disrupting the DNA replication and repair system, leading to bacterial death (Xue et al., 2024). Copper itself also plays an important role in skeletal growth and repair (Hoppe et al., 2013).

Therapeutic window and cytotoxicity risk: Cu is a typical heavy metal ion with a narrow therapeutic window. The oxidative stress and metal toxicity mechanisms that kill bacteria are non-specific. High-concentration Cu leakage from scaffolds induces severe ROS burst in host osteoblasts and PDLCs, causes mitochondrial damage and DNA oxidative injury, and inhibits cell osteogenic differentiation and angiogenesis. Uncontrolled Cu release will lead to significant cytotoxicity and delay periodontal tissue regeneration (Ma et al., 2022; Yu et al., 2024).

#### 3.1.6 Silver (Ag)

Silver (Ag) ions can be incorporated into a variety of bone replacement materials in a number of forms (Newby et al., 2011; Mariappan and Ranga, 2017) and subsequently released throughout the dissolution process. Bacterial growth is restricted through three mechanisms: interference with electron transfer, binding to DNA and contact with cell membranes (Otari et al., 2015).

Therapeutic window and cytotoxicity risk: Ag exhibits the most prominent concentration-dependent cytotoxicity among all six metal ions. Ag ions non-selectively bind to membrane proteins and intracellular genetic substances of host mammalian cells. Excess Ag release induces ROS accumulation, cell cycle arrest, and apoptosis in PDLCs and osteoblasts, severely inhibiting bone regeneration. The conflict between efficient antibacterial activity and host cytotoxicity is the primary bottleneck restricting the clinical translation of Ag-doped periodontal scaffolds, requiring precise controlled-release strategies to balance efficacy and biosafety (Tao et al., 2025).

Summary on the therapeutic window of ions: all six functional metal ions exhibit concentration-dependent dual effects, namely, therapeutic efficacy at low concentrations and host cytotoxicity at high concentrations (Li et al., 2025a). The antibacterial and anti-inflammatory mechanisms of these ions (including reactive oxygen species induction, membrane disruption, protein/DNA damage) lack cell selectivity. While eliminating pathogenic bacteria, excessive dosages will simultaneously injure resident periodontal cells and osteoblasts. To addressQthis challenge, multiple material modification strategies have been proposed (Chen et al., 2021).

Developing sustained or stimulus-responsive delivery systems. Such carriers avoid burst release and maintain ion concentrations within an effective yet non-cytotoxic range. Particularly, pH- or ROS-responsive biomaterials enable on-demand ion release only within infected microenvironments, minimizing off-target toxicity to healthy periodontal tissues. Ionic combinatorial design.; Synergistic antibacterial effects can be achieved using mixtures of metal ions at reduced individual concentrations. Certain ions (e.g., Sr^2+^) simultaneously promote osteogenic differentiation and activate cellular antioxidant signaling, counteracting the oxidative damage induced by antibacterial metal ions. Targeted surface functionalization.; Modifications with bacteria-affinitive moieties enrich metal ions preferentially on bacterial membranes, reducing direct exposure to host cells; Co-delivery of antioxidant agents to elevate the oxidative stress tolerance of osteoblasts and PDL cells, widening the safe concentration range without ablating antibacterial ROS. Nevertheless, most strategies remain at the *in vitro* or preliminary *in vivo* stage. Uniform quantitative thresholds for each ion cannot be generalized due to variations in material degradation, local microenvironment, and cell models. Establishing standardized therapeutic windows for translational periodontal and implant applications still requires systematic further investigation.

### 3.2 Natural anti-inflammatory molecules

Inflammation treatment is mainly based on chemical drugs, including non-steroidal anti-inflammatory drugs (NSAIDs) and glucocorticoids, which have various toxic side effects such as cardiotoxicity, hepatotoxicity and immune dysfunction (Navashin et al., 1977). Compared with non-steroidal anti-inflammatory drugs and glucocorticoids, natural anti-inflammatory drugs have a lesser burden on the liver, gastrointestinal tract and kidney, resulting in side effects such as gastrointestinal ulcers, liver and kidney damage, etc. However, studies have shown that some natural anti-inflammatory drugs, such as resveratrol, can reduce blood cholesterol and triglyceride levels, inhibit platelet aggregation, and thus reduce the risk of cardiovascular disease (Xie et al., 2014; Ye et al., 2019), which shows great advantages compared with the side effects of non-steroidal anti-inflammatory drugs and glucocorticoids on blood pressure and lipids. In addition, compared with the single anti-inflammatory mechanism of NSAIDS inhibiting the synthesis of prostaglandins by inhibiting COX and glucocorticoids (Kurumbail et al., 1996) inhibiting the activation of inflammatory cells and inflammatory mediators, natural drugs have the advantage of multi-target action and can regulate inflammation more comprehensively.

#### 3.2.1 Astaxanthin

Astaxanthin is a natural fat-soluble orange-red carotenoid. It has powerful antioxidant properties, anti-inflammatory, anti-apoptotic and immunomodulatory. It has been widely used to cure a variety of inflammatory diseases, including reducing gouty arthritis by modulating levels of various inflammatory markers (Zhang L. et al., 2021), reducing inflammation of the nervous system (Wu et al., 2015; Liang et al., 2024),and skin, kidney and other related systemic diseases (Kohandel et al., 2022). The anti-inflammatory mechanism of action of astaxanthin mainly involves inflammatory markers and multiple signaling pathways, including PI3K/AKT, Nrf2, NF-κB, ERK1/2, JNK, p38, MAPK and JAK-2/STAT-3 (Chang and Xiong, 2020). Astaxanthin also regulates the cyclooxygenase-2 (COX-2) pathway and reduces and inhibits cytokines and other inflammatory factors such as IL-6 and TNF-α(Ahmadi and Ayazi-Nasrabadi, 2021). Hengfang Zhang et al. prepared chitosan (CS)/polyvinyl alcohol (PVA)/graphene oxide (GO)/astaxanthin (ASTA) nanofiber membranes by electrospinning to improve the periodontal inflammatory microenvironment (Zhang et al., 2021c).

#### 3.2.2 Paeoniflorin

Paeoniflorin (Pae) inhibits inflammatory responses, regulates immune cell function and activation, reduces the production of inflammatory mediators, and repairs aberrant signaling pathways. Pae can achieve a balance of immune cell subsets by inhibiting abnormally activated cell subsets and restoring regulatory cell subsets. Pae can regulate signaling pathways such as MAPK/NF-κB pathway (Li Y. et al., 2022), PI 3 K/Akt pathway (Li Q. et al., 2024), JAK 2/STAT 3 pathway, TGFβ/Smads (Zhou et al., 2013), etc (Ni et al., 2016). Oxidative stress and NLRP-3 inflammastor-mediated pyrodeath can be inhibited through the Nrf 2 signaling pathway (Zhou et al., 2024). Ni J from Wuhan University used paeoniflorin in a periodontitis model and found that paeoniflorin can significantly inhibit the destruction of alveolar bone and soft tissue during the onset of experimental periodontitis (Ni et al., 2016).

#### 3.2.3 Ginkgolide B

Ginkgolide B (GB) is an important bioactive ingredient extracted from the traditional Chinese medicine Ginkgo biloba, Ginkgo biloba leaf extract significantly inhibits NF-κB p65 subunit translocation, IκB-α phosphorylation (Li et al., 2009), IKK-β activity, and downstream inflammatory cytokines and proteins expression (Zhang et al., 2011) mediated by zinc finger protein A20, which can attenuate inflammatory responses through the A20-NF-κB signaling pathway (Zhang et al., 2018). Moreover, ginkgolide B can significantly inhibit LPS-induced MAPK pathway activation (Chu et al., 2011; Hu et al., 2018). This is of great significance for the fight against periodontitis caused by gram-negative bacteria.

#### 3.2.4 Andrographylaxis

Andrographylaxis has a variety of biological activities, including antitumor, antibacterial, anti-inflammatory, antiviral, antifibrotic, anti-obesity, immunomodulatory, and hypoglycemic (Zhang et al., 2021b). Andrographylaxis inhibits NF-κB and STAT 3 activation by interfering with the expression of the SOCS 1 and SOCS 3 signaling pathways, which downregulates inflammatory iNOS and Cox-2 gene expression (Lee et al., 2011).

#### 3.2.5 Triptolide

Triptolide is a bioactive diterpene tricyclic oxide isolated from Lei Gong Teng, a traditional Chinese medicinal plant, whose extracts have been used as anti-inflammatory and immunosuppressive drugs for centuries (Tong et al., 2021). Various extracts inhibit the expression of inflammatory factors, and are mostly used in the treatment of arthritis, cancer, and other diseases (Zhang and Wei, 2020). Chen, H et al. revealed through experiments that the mechanism by which triptolide alleviated the inhibitory effect of TNF-α on the osteogenesis of hPDLSCs may be related to the p-IκBα/NF-κB pathway (Chen et al., 2024).

#### 3.2.6 Tetramethylpyrazine

Tetramethylpyrazine (TMP) the active ingredient in the Chinese medicine Ligusticum chuanxiong, has antioxidant and anti-inflammatory properties. Its anti-inflammatory effects may be mediated by inhibition of the expression of tumor necrosis factor-α (TNF-α), IL-1, IL-10 (Zhang et al., 2020), and prostaglandin E2 (PGE2), especially through inhibition of the activation of NF-κB (Zhang et al., 2020) and the downregulation of cyclooxygenase-2 (COX-2) and inducible nitric oxide synthase (iNOS) (Li and Gong, 2022). According to research, ligustrazine can reduce the inflammatory level and apoptosis of human periodontal ligament cells stimulated by LPS by down-regulating miR-302b (Duan et al., 2020).

#### 3.2.7 Ginsenoside

Ginsenoside (Rb) is a major bioactive saponin in the traditional Chinese medicine ginseng, and Rb1 exerts these effects by regulating mitochondrial energy metabolism, mitochondrial division and fusion, apoptosis, oxidative stress and reactive oxygen species release, mitochondrial autophagy, and mitochondrial membrane potential (Zhou et al., 2019). Ginsenoside Rb1 can be transported to the inflammatory site in multiple ways and shows good therapeutic effect on periodontitis (Guan et al., 2024). In addition, Rd has good antibacterial activity against porphyromonas gingivalis, the main pathogen of periodontitis, by reducing the virulence of Porphyromonas gingivalis and inhibiting its biofilm to play an antibacterial role, and improving the abundance of porphyromonas gingivalis subgingival (Zhou et al., 2022). Most importantly, ginsenoside itself can inhibits osteoclastogenesis via ERK/NF-κB signaling pathway (Sun et al., 2023). This anti-inflammatory and bone damage inhibiting drug properties make Ginsenoside a perfect fit for the disease model of periodontitis, showing great therapeutic potential.

#### 3.2.8 Asiaticoside

Asiaticoside is a triterpenoid found in the traditional Chinese medicine *Centella asiatica* (Bandopadhyay et al., 2023), and has been used in the treatment of a variety of inflammatory diseases due to its excellent anti-inflammatory properties (Chen et al., 2014). Asiaticoside is a natural anti-inflammatory medicine that is used in the treatment of inflammatory diseases. According to studies, asiaticoside can block the production of LPS-induced iNOS, cyclooxygenase (COX-2) and interleukin-1α (IL-1a) expression (Yun et al., 2008) and the LPS-induced inflammation and apoptosis of hPDLSCs can be alleviated through TLR 4/NF kappa B pathway, which is expected to be a therapeutic drug for periodontal tissue regeneration (Zou et al., 2023).

#### 3.2.9 Quercetin

Quercetin is a plant polyphenol with a wide range of biological activities, including antitumor, anti-inflammatory, antiviral, antilipid peroxidation, antiplatelet aggregation, and anticapillary permeability (Li Y. et al., 2016). Potential mechanisms of action of quercetin include regulation of lipopolysaccharide (LPS)-induced production of tumor necrosis factor-α (TNF-α) (Nair et al., 2006), inhibition of inflammation-producing enzymes (cyclooxygenase (COX) (Yang et al., 2021) and lipoxygenase (LOX) (Fiorucci et al., 2008) production. Quercetin inhibits phosphatidylinositol-3-kinase (PI3K)-(p85) tyrosine phosphorylation and Toll-like receptor 4 (TLR4)/MyD 88/PI3K complex formation to limit LPS-induced inflammation, and inhibits Fcε RI-mediated release of pro-inflammatory cytokines, trypsin-like enzymes, and histamine. The team of Professor Hongchang Lai used quercetin encapsulated in bioadhesive mesoporous polydopamine to treat periodontitis (Zhang et al., 2024b). Like most natural drugs, quercetin also has problems such as poor stability, poor water solubility and low bioavailability, which limit its clinical application. However, quercetin can be prepared together with other substances into nanoparticles or nanofibers to deliver quercetin to the periodontitis site to overcome these problems and realize the treatment of periodontitis. For example, Di Cristo, F et al. achieved effective treatment of periodontitis by preparing quercetin and polylactic acid (PLA) into nanofibers (Di Cristo et al., 2022).

#### 3.2.10 Lignans

Lignans inhibits proinflammatory mediators (e.g., IL-1β, IL-6, IL-8, IL-17, IL-22, TNF-α, and COX-2) and modulate inflammatory processes regulated by a variety of signaling pathways (e.g., the NF-κB, JAK-STAT, and TLR signaling pathways (Gendrisch et al., 2021)). Min BS team found two novel ligans isolated from the Saururus chinensis These two new lignans have been experimentally verified to play an anti-inflammatory role by activating Nrf2 and HO-1 and their anti-inflammatory effects by activation of Nrf2 and HO-1 (Min et al., 2016).

#### 3.2.11 Sinomenine

Sinomenine (SIN) is an isoquinoline alkaloid with analgesic, anti-osteolysis, anti-inflammatory and immunosuppressive effects. It reduces the release of inflammatory cytokines such as tumor necrosis factor-α (TNF-α) (Yi et al., 2010), interleukin-6 (IL-6), and IL-1β (Yang and Chen, 2008), thereby attenuating the inflammatory response, and is mostly used in targeted therapy for rheumatoid arthritis. Because sinsinine has properties that inhibit IL-6, PGD (2), LTC 4, β-Hex, and COX-2, it also has potential in the treatment of allergic reactions (Oh et al., 2011). Meanwhile, sinomenine regulates the expression of heme oxygenase-1 and autophagy inhibits inflammation (Peng et al., 2018). SIN’s modulation of the α 7 nAChR and its regulation of the methylation level of a specific GCG locus in the promoter of mPGES-1, as well as its direct targeting of guanylate-binding protein 5 (GBP 5) mechanism, undoubtedly enrich the possibility of SIN for the treatment of inflammatory immune-related diseases (Li et al., 2023).

#### 3.2.12 Berberine

Berberine, an isoquinoline alkaloid from the Chinese herb Huanglian, counteracts oxidative stress and inflammation through a very complex mechanism consisting of multiple kinases and signaling pathways involving a variety of factors, including NF-κB (nuclear factor-κB) (Li Z. et al., 2016) and AMPK (Han et al., 2010) (AMP-activated protein kinase). In addition, MAPK (mitogen-activated protein kinase) and Nrf 2 (nuclear factor erythroid-2-related factor 2) are also involved in the mechanisms of oxidative stress and inflammation (Ma et al., 2018). Periodontitis can be effectively treated by achieving local controllable drug release through incorporation into a thermosensitive hydrogel (Wang et al., 2023d). Wang Chang et al. prepared berberine thermosensitive hydrogel to treat periodontitis. Experiments showed that berberine loaded hydrogel significantly increased the level of osteogenic factors, and its anti-inflammatory osteogenic effect may be realized through the PI 3K/AKT signaling pathway (Wang et al., 2023d).

#### 3.2.13 Gallocatechin gallate

Gallocatechin gallate (EGCG) is a strong antioxidant compound from green tea. Due to its multiple interactions with the inhibition of NLRP3 inflammasome activation (Zhang C. et al., 2021) and heme oxygenase-1 (H0-1) (Mokra et al., 2022), cell surface receptors, intracellular signaling pathways, and nuclear transcription factors conferring anti-inflammatory (Liang et al., 2019), antioxidant, antifibrotic, antiremodeling, and tissue-protective properties to EGCG, it has demonstrated considerable medical value in the treatment of periodontitis (Mokra et al., 2022). EGCG NPs have a great potential for ROS scavenging and remodeling of the macrophage by inducing polarization from the M1 to the M2 phenotype in the inflammatory microenvironment for effective periodontitis treatment (Tian et al., 2022). Qingchen Feng et al. developed a hydrogel repair system based on ROS/glucose-stimulated responsive EGCG release to effectively treat periodontitis (Feng et al., 2024). The scaffold was designed to simultaneously regulate the mechanical properties of the OSA-PBA/CMC + EGCG hydrogel (OPCE) by using an injectable hydrogel loaded with phenoborate functionalized sodium alginate (OSA-PBA) and carboxymethyl chitosan (CMC) for gallatechin-3-gallate (EGCG). The hydrogel has demonstrated unique adaptive, antioxidant and anti-inflammatory properties, injectable and ROS/glucose-triggered EGCG release, making it an ideal drug delivery vehicle and bone repair material.

#### 3.2.14 Resveratrol

Resveratrol is a natural polyphenol and the molecular basis of its pleiotropic activity is based on its ability to modulate a wide range of cell signaling molecules, said cell signaling molecules being, for example, HSP 70,COX-2, nuclear factor-κB (NF-κ B) (Elmali et al., 2005; Bishayee et al., 2010), interleukin-1 beta (IL-1 beta), tumor necrosis factor-alpha (TNF-alpha)) (Wang L. et al., 2023), matrix metalloproteinases (Amontree et al., 2024), Wnt (Chen et al., 2012), Notch (Wang L. et al., 2023), AMP-activated protein kinase (Park et al., 2007), vascular cell adhesion molecules, intercellular adhesion molecules (Deng et al., 2011), silent information regulator 1(SIRT1)-related targets including peroxisome proliferator-activated receptor gamma coactivator 1-alpha (PGC-1 alpha), nuclear factor erythroid2-related factor 2 (Nrf2), high mobility group box 1 protein (HMGB1), NOD-like receptor thermal protein domain associated protein 3 (NLRP3), p38/p53, as well as endothelial nitric oxide synthase (eNOs) (Singh et al., 2019; Liu et al., 2024). Inhibition of Toll-like receptor (TLR)[114] and pro-inflammatory gene expression Targeted silencing of regulatory proteins, adenosine monophosphate kinase, nuclear factor-κB, inflammatory cytokines, and antioxidant enzymes along cellular processes such as angiogenesis, lipid metabolism, mitochondrial biogenesis, vasculogenesis, and apoptosis (Malaguarnera, 2019). It can act as a coating molecule or a nanoparticle effector molecule (Bhattarai et al., 2016). Table 1 presents the mechanisms of action of the above‐mentioned natural anti‐inflammatory drugs.

**TABLE 1:** This is a table summarizing the mechanism of action of the above natural anti-inflammatory drugs.

Medicine	Anti-inflammatory mechanism	Reference
Astaxanthin	Regulates the cyclooxygenase-2 (COX-2) pathway and cytokines and other inflammatory factors such as IL-6 and TNF-α	(Chang and Xiong, 2020) (Ahmadi and Ayazi-Nasrabadi, 2021)
Paeoniflorin	Regulate MAPK/NF-κB pathway, PI3K/Akt pathway, JAK2/STAT3 pathway, TGFβ/Smads pathway, oxidative stress and NLRP-3 through the Nrf 2 signaling pathway	(Li et al., 2022b) (Li et al., 2024b) (Zhou et al., 2013) (Ni et al., 2016) (Zhou et al., 2024) (Ni et al., 2016) (Zhou et al., 2024)
Ginkgolide B	Regulate NF-κB p65 subunit translocation, IκB-α phosphorylation, IKK-β activity, inflammatory cytokines and proteins expression mediated by zinc finger protein A20, and LPS-induced MAPK pathway activation	(Li et al., 2009; Chu et al., 2011; Zhang et al., 2011 , 2018; Hu et al., 2018)
Andrographylaxis	Regulate NF-κB and STAT3 activation by interfering with the expression of the SOCS 1 and SOCS 3 signaling pathways, which downregulates inflammatory iNOS and Cox-2 gene expression	(Lee et al., 2011)
Triptolide	Regulate the expression of TNF-αrelated to the p-IκBα/NF-κB pathway	(Zhang and Wei, 2020) (Chen et al., 2024)
Tetramethylpyrazine	Regulate the expression of TNF-α, IL-1, IL-10, PGE2, NF-κB, COX-2 and iNOS.	(Zhang et al., 2020) (Zhang et al., 2020) (Li and Gong, 2022)
Ginsenoside	Regulate mitochondrial energy metabolism, mitochondrial division and fusion, apoptosis, oxidative stress and reactive oxygen species release, mitochondrial autophagy, and mitochondrial membrane potential	(Zhou et al., 2019) (Guan et al., 2024) (Zhou et al., 2022) (Sun et al., 2023)
Asiaticoside	Regulate the production of LPS-induced iNOS, cyclooxygenase (COX-2) and interleukin-1α(IL-1a) expression	(Yun et al., 2008) (Zou et al., 2023)
Quercetin	Regulate TNF-α, COX, LOX, PI3K-AKT signal pathway and LPS signal pathway	(Nair et al., 2006) (Yang et al., 2021) (Fiorucci et al., 2008) (Zhang et al., 2024b)
Lignans	Regulate IL-1β, IL-6, IL-8, IL-17, IL-22, TNF-α, COX-2 and the NF-κB, JAK-STAT, and TLR signaling pathways	(Min et al., 2016; Gendrisch et al., 2021)
Sinomenine	Regulate TNF-α, IL-6, IL-1β, PGD(2), LTC4, β-Hex, and COX-2, heme oxygenase-1 and autophagy	(Yi et al., 2010) (Yang and Chen, 2008) (Oh et al., 2011) (Peng et al., 2018) (Li et al., 2023)
Berberine	Regulate NF-κB, AMPK and PI3K/AKT signaling pathway	(Li et al., 2016b) (Han et al., 2010) (Ma et al., 2018) (Wang et al., 2023d)
Gallocatechin gallate	Regulate NLRP3 inflammasome activation, heme oxygenase-1 (H0-1), cell surface receptors, intracellular, signaling pathways, nuclear transcription factors Remodel the macrophage by inducing polarization from the M1 to the M2 phenotype	(Zhang et al., 2021a) (Mokra et al., 2022) (Liang et al., 2019) (Tian et al., 2022)
Resveratrol	Regulate HSP 70,COX-2,NF-κ B,IL-1 beta,TNF-α, matrix metalloproteinases,Wnt,Notch,AMP activated protein kinase,vascular cell adhesion molecules, intercellular adhesion molecules, SIRT1-related targets including peroxisome proliferator-activated receptor gamma coactivator 1-alpha (PGC-1 alpha), nuclear factor erythroid2-related factor 2 (Nrf2), high mobility group box 1 protein (HMGB1), NOD-like receptor thermal protein domain associated protein 3 (NLRP3), p38/p53, eNOs, TLR	(Elmali et al., 2005) (Bishayee et al., 2010) (Wang et al., 2023b) (Chen et al., 2012) (Park et al., 2007) (Deng et al., 2011) (Singh et al., 2019) (Liu et al., 2024)

## 4 Material treatment for anti-inflammatory

### 4.1 Material surface structure treatment

The surface properties of biomaterials (surface morphology, roughness, porosity, *etc.*) can influence protein adhesion, and the protein layer formed can further mediate the state of the coagulation system, complement system, platelets, and immune cells, as well as further recruitment and adhesion (Batool et al., 2021). Therefore, for the surface morphology and state of the bone substitute material, it is important to alter the surface charge, wettability, stiffness, adhesion points, and mechanical interactions of the material to resist bacterial adhesion from the initial stages of inflammation (Li W. et al., 2021).

For example, for bioglasses inorganic substances can be engineered into the glass network or surface modifications can be performed after glass formation. In addition, different temperature schemes can be used to induce phase separation. This phase separation can produce specific groups of ionic components that are released at different rates, thereby increasing biological properties. On the other hand, the glass can be subjected to controlled heat treatment, thereby producing glass-ceramic biomaterials with different properties. Different properties can be obtained by inducing crystalline phase formation or by forming residual glass in the glass-ceramic structure, thus providing different release profiles (Manivasagan et al., 2024).

For implant surfaces, bioactive coatings with different compositions can be prepared by means of plasma treatment and adjusting radio frequency current and gas atmosphere to achieve antibacterial effects (Chen M. et al., 2022). Moreover, the surface structure of materials can also be combined with photothermal therapy and other methods to promote the effect of photothermal therapy. For example, a special bowl-shaped surface structure is prepared to increase the amount of light reflection to enhance antibacterial performance (Wu et al., 2022). And more and more surface structures combine bionics principles. For example, the team led by Academician Ren Luquan of Jilin University has prepared an adaptive surface through the nanostructure inspired by cicada wings to achieve dual functions of antibacterial and tissue integration, showing objective prospects (Liu et al., 2022).

### 4.2 Photothermal coatings

Photothermal therapy (PTT) uses a photothermal agent (PTA) to convert light energy into heat to generate localized hyperthermia to eliminate bacteria (Chen et al., 2020). PTT can disrupt the intact structure of bacteria by non-invasively generating high temperatures in their biologically active matrices (e.g., proteins and nucleic acids), allowing antimicrobial agents to penetrate more readily and eradicate protected bacteria, and PTT prevents bacterial drug resistance by simultaneously targeting multiple cellular components at the same time (He et al., 2023).

However, the lack of specificity of photothermal therapy and the lack of laser penetration limit its development in the antimicrobial field. Chemodynamic therapy (CDT) is an emerging antimicrobial therapeutic strategy that utilizes Fenton-/fenton-like metal-based nanocatalysts to convert hydrogen peroxide (H2O2) into hydroxyl radicals (OH) to eradicate Multiple Drug Resistance (MDR) bacterial infections. Despite its great potential, CDT alone has limitations, such as low catalytic potency and insufficient H2O2 generation (Manivasagan et al., 2024). In addition, CDT can also be used as a catalyst for the treatment of MDR bacterial infections.

Combining two methods can achieve better results. For example, a spiky gold-manganese oxide nanozyme developed by Xiaonan Li and Siman Luo et al. that fully exerts the combined antibacterial effect of PTT and CDT can effectively kill a variety of bacteria (Li X. et al., 2022).

### 4.3 Photodynamic coating

PDT is a cold photochemical reaction that utilizes a combination of photoactive chemicals photosensitizers and appropriate wavelengths of light to produce reactive oxygen species (ROS) and subsequent cytotoxicity. When photosensitizer is added to normal tissues and cells, the photosensitizer will be absorbed and cleared after a period of time; abnormal tissues or cells in contact with the photosensitizer will retain the photosensitizer and activate the photosensitizer under the excitation of appropriate wavelengths of light, transferring the light energy by photochemical reaction to the substances in the tissues to produce cytotoxic oxygen radicals or some oxidatively active molecules to attack the cellular structure, causing damage to target cells or bacteria, so as to kill abnormal cells and achieve cytotoxicity. Damage to the target cells or bacteria, thus achieving the effect of killing abnormal cells or bacteria. Emerging nanophotosensitizers include nanoparticles, polymers, lipids, graphene, and other carbon-based nanomaterials (Youf et al., 2021). The construction of PDT coatings with high ROS yields is beneficial in preventing implantation of infections (Gnanasekar et al., 2023).

## 5 Composite bioceramic scaffold materials with anti-inflammatory effects

Hydroxyapatite, calcium triphosphate, and bioglass-ceramics are common bone-substituting materials used in orthopedics and dentistry that exhibit good biocompatibility, bone-enhancing properties, and some of the materials themselves show good antibacterial and anti-inflammatory potential. Multifunctional composites made by combining with additional materials show a wide range of applications and prospects.

### 5.1 Hydroxyapatite

Hydroxyapatite [HA, Ca10(PO4)6(OH)2] is a good bone substitute material. Its surface characteristics are highly consistent with autologous hydroxyapatite (Peng et al., 2011). The sintered HA has excellent stability but this also leads to its inefficient degradation *in vivo* (Renooij et al., 1985).

It has been shown that HA promotes bone growth on the surface of biomaterials and induces the differentiation of progenitor cells to osteoblasts. The mechanism may be that calcium and phosphate ions released from HA scaffolds contribute to osteoblast differentiation, accelerate bone formation through calcification, and upregulate the expression of genes related to osteoblast differentiation (Mondal et al., 2023). The particle size of HA has a great influence on its biological properties, anisotropically regulating immune cell effector function, with needle- and micrometer-sized particles preferentially driving the inflammatory monocyte and macrophage phenotypes and producing proinflammatory cytokines such as TNFα, IL-6, and IL-1β. Nano-hydroxyapatite has the ability to promote anti-inflammatory macrophage phenotypes and exert bone-enhancing effects on mesenchymal stem cells (MSCs) (Shanley et al., 2023).

This brings new perspectives on material improvement and development for HA.

### 5.2 TCP

β-TCP is a high-temperature phase usually obtained by thermal transformation of amorphous calcium phosphate or calcium-deficient hydroxyapatite (Ca9(PO4)5(HPO4)OH; CDHA) at temperatures above 650 °C–750 °C. The phase is usually formed by sintering the calcium phosphate or calcium-deficient hydroxyapatite. Two isomers, α-TCP and β-TCP, can be obtained from different sintering temperatures (Bohner et al., 2020). The isomers of α-TCP and β-TCP have been shown to have a higher sintering temperature than HA.

Compared with HA, TCP is more easily degraded and has higher solubility. *In vitro* and *in vivo* experiments and clinical studies have shown that β-TCP has good biocompatibility, osteoconductivity, and osteogenicity (Shao et al., 2016) and can be directly integrated with primary bone without triggering inflammatory reactions, toxic side effects, and foreign body reactions (Peng et al., 2011).

### 5.3 BG

In physiologic fluids, a layer of hydroxycarbonate apatite similar to bone mineral forms on the surface of bioglass45 S5. Collagen from surrounding tissues is adsorbed onto this layer, attracting osteoblasts and favoring bone regeneration. Thus, bioglass is an osteoinductive material (Abdollahi et al., 2013). BGs show great potential in antibacterial, anti-inflammatory, and osteoinductive properties. BGs significantly reduced the cellular expression of several inflammatory factors such as IL-1β, IL-6, TNF-α, and NO. BGs can manage the expression of key proteins in the NF-κB signaling pathway. BGs are locally weakly alkaline and release Si and Ca ions when in contact with body fluids, creating a special microenvironment. This may be related to the *in vitro* and *ex vivo* inhibitory effects of BGs on the TLR 4/MyD 88/NF-κ B signaling pathway inhibition (Wang W. et al., 2023).

The degradation of the intrinsic network of the bioactive glass with the effect of the degraded components on the surrounding environment leads to the fact that the bioactive glass without any bactericidal ions exhibits bactericidal properties against a fraction of the bacteria in a concentration-dependent form, which is mechanistically related to the pH versus the ion concentration. The adhesive capacity of glass-ceramic biomaterials relies on the degradation process of the biomaterial and the subsequent formation of an HA layer on its surface, which mimics the mineral bone component and bonds firmly to living bone tissue. Briefly, the method follows the steps of (1) ion dissolution from the glass into the medium, (2) reaction of dissolved Ca2+ with (PO4)3- from the medium and subsequent precipitation of an amorphous calcium phosphate (ACP) layer, (3) increase in pH imbalance and ion dissolution to support the growth of the ACP, and (4) crystallization of the ACP layer into an HA layer by combining (OH)- and (CO3)2-(Li J. et al., 2024).

### 5.4 BCP

Bidirectional calcium phosphate ceramic materials consisting of different ratios of hydroxyapatite and β-tricalcium phosphate are widely used in dental bone restoration works due to their excellent physicochemical properties and biocompatibility.

BCP ceramics with a range of different composition ratios tend to produce similar and occasionally opposite results. Overall, when the HA ratio is higher, the material degrades more slowly and absorbs more stably; when the β-TCP ratio is higher, more of the connective tissue and angiogenesis promoting properties of β-TCP are demonstrated (Shao et al., 2022).

Activation of calcium channels in macrophages by biphasic calcium phosphate (BCP) leads them to release the chemokines Ccl3 and Ccl17 to recruit CD4^+^ T cells, mainly regulatory T cells (Tregs), which play a crucial role in ectopic osteogenesis by BCP(Li J. et al., 2024).

To systematically compare the intrinsic properties of these four representative ceramic biomaterials, Table 2 summarizes their degradation behavior, mechanical characteristics, innate biological activity, advantages, limitations and commonly adopted modification strategies.

**TABLE 2:** Summary of key characteristics and modification strategies of typical anti-inflammatory bioceramic scaffolds.

Material	Degradation rate	Mechanical properties	Innate biological activity	Core advantages	Main limitations	Representative modification strategies for anti-inflammatory periodontal application
HA	Slow	High compressive strength; brittle	Promotes osteogenic differentiation; particle-size-dependent macrophage polarization	Excellent biocompatibility; biomineralization ability; stable volume retention	Slow degradation limits material remodeling; micron HA may induce pro-inflammatory response	Nano-crystallization; composite with natural anti-inflammatory phytomolecules; ion doping (Sr, Zn)
β-TCP	Moderate–fast	Lower mechanical strength than HA; poor mechanical stability under load	Osteoconductive; promotes angiogenesis	Fast degradation matching bone turnover; low inflammatory risk	Rapid degradation may cause volume collapse; insufficient mechanical support	Composite with HA to fabricate BCP; loading anti-inflammatory agents; polymer blending
Bioactive glass (45S5)	Fast	Low mechanical strength; fragile	Osteoinductive; intrinsic antibacterial activity; suppresses TLR4/NF-κB pathway	Ion microenvironment regulation; rapid surface apatite formation; dual anti-inflammatory/antibacterial effects	Poor mechanical performance; fast degradation induces drastic pH fluctuation	Ion doping; composite with HA/polymer; immobilization of natural anti-inflammatory compounds
BCP (HA/β-TCP mixture)	Tunable (depended on HA/β-TCP ratio)	Tunable mechanical properties between HA and β-TCP	Immunomodulation; recruits Treg cells; supports osteogenesis	Degradation and mechanical properties are customizable; balanced immune response	Property optimization requires precise control of phase ratio	Adjustment of HA/β-TCP ratio; incorporation of bioactive small molecules; pore structure engineering

Degradation rate and mechanical performance are summarized based on conventional sintered porous scaffolds. The biological behavior varies greatly with porosity, grain size, doping ions and surface modification.

The above four commonly used bone substitute materials are widely used in the treatment of periodontitis due to their superior biological properties and mechanical strength. Compared with simple composite construction with antibiotics to endow materials with antibacterial and anti-inflammatory properties, the above-mentioned treatment methods such as natural anti-inflammatory drugs are used for composite construction of materials, such as preparation, to achieve endowing these four simple materials with better antibacterial and anti-inflammatory properties while avoiding the risks brought by antibiotics. It provides a certain reference for the future development direction of materials.

## 6 Discussion

Periodontitis, as a chronic inflammatory oral disease characterized by irreversible alveolar bone resorption, poses a severe challenge to clinical alveolar bone defect repair. The core dilemma of traditional bone substitute material application in periodontitis treatment lies in the failure to adapt to the local pathological microenvironment with persistent bacterial infection and chronic inflammation, which easily leads to implantation failure, while the addition of antibiotics not only induces bacterial resistance but also causes systemic toxic and side effects. This review takes the synchronous realization of antibacterial, anti-inflammatory and osteogenic triple functions as the core design concept of bioceramic bone tissue-engineered substitutes for periodontitis treatment, and systematically sorts out the inflammatory regulatory mechanism of periodontitis, the screening of bioactive anti-inflammatory molecules and the modification strategy of bioceramic materials, which provides a multi-dimensional theoretical and technical basis for the development of novel bone graft materials suitable for the pathological characteristics of periodontitis.

The occurrence and development of periodontitis-associated alveolar bone resorption is a complex biological process regulated by multiple interrelated inflammatory signaling pathways and inflammasome mechanisms. The cGAS-STING, NF-κB, JAK-STAT and other signaling pathways form a complex regulatory network in the periodontal inflammatory microenvironment, and their abnormal activation is the core molecular basis of sustained inflammation and osteoclast overactivation. Meanwhile, the RANKL/RANK/OPG system, as the final link of alveolar bone remodeling, is the key target for balancing osteogenesis and bone resorption in periodontitis treatment. The in-depth elaboration of these molecular mechanisms in this review clarifies the core target of material modification: the ideal anti-inflammatory and osteogenic bioceramic material should not only block the abnormal activation of inflammatory signaling pathways to alleviate local chronic inflammation, but also regulate the osteogenesis-osteolysis balance through the key osteogenic signaling pathways, which provides a molecular-level guidance for the precise screening of bioactive molecules and the directional modification of materials.

The screening and application of bioactive anti-inflammatory molecules are the core links to endow bioceramic materials with antibacterial, anti-inflammatory and osteogenic multifunctions. Metal ions represented by Sr, Zn, Mn, Mg, Cu and Ag exert their biological effects through multiple mechanisms: on the one hand, they inhibit bacterial growth through physical and chemical effects such as destroying bacterial cell membranes and interfering with intracellular metabolism, and on the other hand, they regulate immune cell polarization and inhibit the activation of inflammatory signaling pathways to alleviate inflammation, while most metal ions can also directly promote osteoblast proliferation and differentiation and inhibit osteoclast activity, realizing the integration of “antibacterial-anti-inflammatory-osteogenic” effects. Natural anti-inflammatory molecules such as astaxanthin, paeoniflorin and ginsenoside have the advantages of multi-target action, mild effect and low metabolic burden compared with chemical anti-inflammatory drugs such as non-steroidal anti-inflammatory drugs and glucocorticoids. They can regulate multiple inflammatory signaling pathways at the same time, scavenge reactive oxygen species, and even improve the local microenvironment of periodontitis by regulating macrophage polarization (M1→M2). However, natural anti-inflammatory molecules have inherent defects such as poor stability, low water solubility and low bioavailability, which limit their direct application in material modification. The preparation of nanoparticles, nanofibers and other composite delivery systems is an effective way to solve this problem, and it is also an important research direction for the subsequent optimization of natural active molecule application.

Material modification strategies based on surface engineering and functional coating construction further improve the adaptability of bioceramic materials to the periodontal inflammatory microenvironment. The surface morphology, roughness and porosity of materials directly affect bacterial adhesion and immune cell response. Bionic surface structure design (such as cicada wing-inspired nanostructure) and plasma surface treatment can reduce bacterial initial adhesion from the source and promote the adhesion and proliferation of osteoblasts and periodontal ligament cells, realizing the dual functions of antibacterial and tissue integration (Li et al., 2025b, 2026). Photothermal coating and photodynamic coating construct a non-antibiotic antibacterial system: photothermal therapy generates local hyperthermia to kill bacteria through light energy conversion, and photodynamic therapy produces reactive oxygen species to induce bacterial apoptosis, both of which can effectively avoid bacterial resistance. The combination of photothermal/photodynamic therapy with chemodynamic therapy can form a synergistic antibacterial effect, which significantly improves the antibacterial efficiency of materials in the anaerobic microenvironment of periodontitis. In addition, the surface modification of bioceramic materials can also regulate the release rate of active molecules, realize the sustained and controlled release of metal ions and natural anti-inflammatory molecules, and ensure the long-term antibacterial and anti-inflammatory effects of materials in the process of alveolar bone repair.

Hydroxyapatite (HA), tricalcium phosphate (TCP), bioglass (BG) and biphasic calcium phosphate (BCP) are the most commonly used bioceramic bone substitute materials in clinical dentistry, and their inherent characteristics lay a good foundation for multifunctional modification. HA has good biocompatibility and osteoconductive property, and the regulation of its particle size can realize the transformation of immune regulation effect (from pro-inflammatory to anti-inflammatory), which provides a new idea for its optimization; TCP has higher degradability and solubility than HA, and β-TCP can be directly integrated with native bone without triggering inflammatory and foreign body reactions, which is an ideal matrix material for composite modification; BG has inherent osteogenic induction, antibacterial and anti-inflammatory properties, and its surface can form a hydroxycarbonate apatite layer similar to bone mineral in physiological fluid, which can firmly bond with living bone tissue, and the release of Si and Ca ions can construct a local weak alkaline microenvironment to inhibit the activation of inflammatory signaling pathways; BCP can flexibly regulate its degradation rate and biological properties by adjusting the ratio of HA and β-TCP, and its ability to recruit regulatory T cells (Tregs) provides a new perspective for the immunoregulatory repair of alveolar bone defects. The composite modification of these four materials with metal ions, natural anti-inflammatory molecules and functional coatings can make up for the defects of single material (such as slow degradation of HA, single function of TCP) and realize the integration of triple functions, which is the main development direction of bioceramic materials for periodontitis treatment at present. In this review, we provide a comprehensive list of improved and routinely introduced materials for four clinically utilized materials, as well as an in-depth description of the intrinsic mechanisms of the disease, which will help to inspire the future direction of material development at both the mechanistic and molecular levels.

Although this review systematically combs the molecular mechanism of periodontitis inflammation and the modification strategy of bioceramic materials, there are still certain limitations in the research scope. This review mainly focuses on the traditional bioceramic materials commonly used in clinical practice, and does not involve the research progress of novel composite materials such as electromagnetic bioceramics and hydrogel-based bioceramic composites, which have shown good application potential in bone tissue engineering in recent years. In addition, the current research on modified bioceramic materials is mostly in the *in vitro* cell experiment and animal model stage, and the clinical transformation still faces many challenges: the pathological microenvironment of human periodontitis is more complex (such as the coexistence of multiple pathogenic bacteria, individual differences in inflammatory response), and the degradation rate, active molecule release rate and biological safety of materials in the human body need to be further verified by large-sample and long-term clinical trials. At the same time, the personalized design of materials for different degrees of alveolar bone defects and different inflammatory states is also a key problem to be solved in clinical transformation.

In the future, the research and development of bioceramic bone tissue-engineered substitutes for periodontitis treatment should focus on the following directions: first, precision modification based on the periodontal pathological microenvironment, combining multi-omics technology to further clarify the key molecular targets of periodontitis with different clinical phenotypes, and realizing the directional design of materials; second, optimization of the composite system of active molecules, developing a more efficient delivery system to solve the defects of natural anti-inflammatory molecules and realize the synergistic effect of multiple active molecules; third, construction of intelligent responsive materials, designing materials with responsive release of active molecules to the periodontal inflammatory microenvironment (such as ROS, pH responsive), so as to realize the “on-demand” antibacterial and anti-inflammatory effects; fourth, accelerating the clinical transformation process, combining clinical research to optimize the material preparation process and evaluate the long-term safety and effectiveness of materials, so as to make the modified bioceramic materials better meet the clinicaltreatment needs of periodontitis.

In conclusion, the multifunctional composite bioceramic material integrating antibacterial, anti-inflammatory and osteogenic functions is the development trend of bone substitute materials for periodontitis treatment. Based on the in-depth understanding of the molecular mechanism of periodontitis inflammation, through the precise screening of bioactive anti-inflammatory molecules and the innovative design of material modification strategies, the bioceramic materials can be endowed with the ability to adapt to the periodontal inflammatory microenvironment, which not only eliminates bacterial infection and alleviates chronic inflammation, but also actively promotes alveolar bone defect repair. This review clarifies the core design concept of multifunctional integration of bioceramic materials for periodontitis treatment, and provides a valuable reference for the research and development of novel clinical transformable bone graft materials, which is of great significance for improving the clinical effect of alveolar bone defect repair in patients with periodontitis.
